# Cobblestone-Area Forming Cells Derived from Patients with Mantle Cell Lymphoma Are Enriched for CD133^+^ Tumor-Initiating Cells

**DOI:** 10.1371/journal.pone.0091042

**Published:** 2014-04-10

**Authors:** Daniel J. Medina, Jeneba Abass-Shereef, Kelly Walton, Lauri Goodell, Hana Aviv, Roger K. Strair, Tulin Budak-Alpdogan

**Affiliations:** 1 Department of Medicine, Rutgers - The State University of New Jersey, Robert Wood Johnson Medical School, Rutgers Cancer Institute of New Jersey, New Brunswick, New Jersey, United States of America; 2 Department of Pathology, Rutgers - The State University of New Jersey, Robert Wood Johnson Medical School, Rutgers Cancer Institute of New Jersey, New Brunswick, New Jersey, United States of America; University of Navarra, Center for Applied Medical Research, Spain

## Abstract

Mantle cell lymphoma (MCL) is associated with a significant risk of therapeutic failure and disease relapse, but the biological origin of relapse is poorly understood. Here, we prospectively identify subpopulations of primary MCL cells with different biologic and immunophenotypic features. Using a simple culture system, we demonstrate that a subset of primary MCL cells co-cultured with either primary human mesenchymal stromal cells (hMSC) or murine MS-5 cells form in cobblestone-areas consisting of cells with a primitive immunophenotype (CD19−CD133+) containing the chromosomal translocation t (11;14)(q13;q32) characteristic of MCL. Limiting dilution serial transplantation experiments utilizing immunodeficient mice revealed that primary MCL engraftment was only observed when either unsorted or CD19−CD133+ cells were utilized. No engraftment was seen using the CD19+CD133− subpopulation. Our results establish that primary CD19−CD133+ MCL cells are a functionally distinct subpopulation of primary MCL cells enriched for MCL-initiating activity in immunodeficient mice. This rare subpopulation of MCL-initiating cells may play an important role in the pathogenesis of MCL.

## Introduction

Mantle cell lymphoma (MCL) is a distinct subtype of non-Hodgkin's lymphoma (NHL) that accounts for 5–7% of all NHL cases in the US and Europe [1]–[4]. Most MCL (75%) patients are diagnosed with advanced, stage III/IV disease. Patients often present with extensive lymphadenopathy and extranodal involvement [5]. MCL often has the adverse features of both indolent (incurable) and aggressive (rapidly growing) lymphomas [6]. Despite the usually aggressive nature of the disease, several studies have identified a small subgroup of patients (10–15%) with indolent disease who survive more than 10 years [6]. However, most cases (70–85%) follow a clinical course with comparatively rapid disease progression [2], [6]. The development of more aggressive and targeted therapies has improved the medium survival from 2–3 years to 5–7 years [5]. Despite improving treatment regimens, most patients treated with standard therapies relapse. Once relapse occurs, patients often enter a vicious cycle of treatment followed by relapse, with the time to relapse decreasing with each treatment. This continuous cycle of treatment response followed by relapse indicates that a subset of MCL cells have the capacity to survive treatment and act as a reservoir for subsequent tumor growth. The characteristics of the MCL cells comprising the reservoir are unknown, but may be due in part to MCL cells with stem cell/progenitor cell-like activity [7]–[10].

There is increasing evidence that many cancers contain a small subset of cells with stem cell-like properties often referred to as cancer stem cells (CSCs) or tumor-initiating cells (TICs) [11]–[14]. In many model systems, TICs survive cytotoxic treatment due to their intrinsic resistance to most therapeutic modalities [15]–[19]. TICs can self-renew to generate additional TICs and also differentiate into phenotypically diverse cancer cells to repopulate the tumor cell types found in the bulk tumor [20]. Hence, TICs might explain some recurrences after chemotherapy. Since current cancer therapeutics have been developed to kill differentiated cancer cells, some intrinsically resistant cancer cells (e.g. TICs) may survive these treatments and act as “seeds” for future relapse.

We recently developed a reliable system for long-term culture of primary MCL cells ex vivo [21]. In this system we co-culture MCL cells with murine (MS-5 cells) or human primary mesenchymal stromal cells (hMSC). While studying MCL-hMSCs interactions, we noted the presence of clusters of small lymphoid-like cells under the mesenchymal stem cell layer during long-term co-culture. These clusters are comparable to cobblestone area forming cells (CAFCs) seen when bone marrow (BM) stromal cells are co-cultured with hematopoietic stem cells (HSCs) [22], [23]. It has been reported that the dormant and more primitive hematopoietic cells preferentially migrate beneath the adherent stromal layer, while the cells that migrate to the surface of the layer show increased proliferation and maturity and then shed into the medium [24]–[26]. Analysis of this CAFC MCL cell population showed that these clusters contain self-renewing cells with the chromosomal translocation t(11;14)(q13;q32) characteristic of MCL. Yet these cells have a unique immunophenotype, namely the expression of the HSC marker CD133 and loss of CD19 expression. In this report we present experiments that demonstrate that only this CD19−CD133+ subpopulation of primary MCL cells can self renew and engraft immunodeficient mice.

## Materials and Methods

### Patient specimens and cell culture

Samples from six MCL patients (samples UPN1-UPN6) were included in this study. Diagnosis was based on the immunophenotype (CD5+, CD19+, and CD23−) of the malignant cells in conjunction with expression of cyclin D1 and/or detection of the translocation t(11;14) by FISH analysis. Samples of blood or tissue were obtained under an exemption protocol approved by the University of Medicine and Dentistry of New Jersey Institutional Review Board. MCL patients had active disease at the time of sampling. Because the protocol prohibits collection of any identifying information, the samples collected were only accompanied by diagnosis, and no information regarding disease stage, treatment history or treatment response was available. Mononuclear cells were isolated from 10 to 25 ml of fresh heparinized blood or tissue biopsy by Histopaque centrifugation. Isolated cells were counted, resuspended in freezing medium (90% fetal bovine serum and 10% dimethylsulfoxide), and stored in liquid nitrogen until use.

The mouse stromal cell line MS-5 and human primary bone-marrow stromal cells (i.e. human MSCs; Lonza, Walkersville, MD) were maintained as previously described [21]. Human MSCs were used from passage 3–6. Umbilical cord blood (UCB) derived CD133+cells and UCB B-cells were purchased from StemCell Technologies. CD5+CD19+ B-cells were isolated from UCB B-cells by using anti-CD5 magnetic beads All cells were maintained in A10 medium consisting of α-MEM supplemented with 1 mM sodium pyruvate, 1× non-essential amino acids, 1× pen/strep and 50 µM 2-mercaptoethanol (Invitrogen, Carlsbad, CA) and 10% FBS (Sigma).

### Antibodies and flow cytometry

Antibodies directed against the following human cell surface proteins were obtained from commercial sources: CD45-PC7, CD45-FITC, CD3-PE, CD5-ECD, CD34-FITC, CD19-APC, CD23-PE (Beckman Coulter, Miami, FL) CD133-PE and CD133-APC (Miltenyi Biotec, Auburn, CA). Stained samples were washed in PBS containing 2% FBS and 7-aminoactinomycin (7-AAD). A minimum of 20,000 cells (unless noted otherwise) was analyzed on a Coulter Cytomics FC500 flow cytometer (Beckman Coulter, Miami, FL). All analysis of normal and lymphoma cells were gated on human CD45+ cells. Sequential gates were set to include only viable cells and quadrant markers were set to exclude at least 99.9% of cells labeled with the appropriate fluorochrome labeled isotype controls.

### Cell depletion and MCL fractionation

Frozen samples were thawed, washed twice in PBS, resuspended in A10 medium and evaluated for cell number and viability by trypan blue exclusion. In order to facilitate the enrichment of the CD45+CD19+CD133− and CD45+CD19−CD133+ subpopulations the samples were depleted of unwanted cells (T-cells, CD34+ cells, monocyte/macrophages, endothelial, NK and thrombocytes) by using a cocktail of lineage specific (CD2, CD3, CD11b, CD14, CD16 CD34, CD56) biotin-conjugated antibodies and anti-biotin magnetic beads followed by separation on two sequential columns on an autoMACS Pro Separator (Miltenyi Biotec, Auburn, CA). The negative fraction containing the unlabeled CD19− and CD19+ cells were collected, washed and resuspended in A10 medium. This was followed by additional fractionation into the CD45+CD19+CD133− and CD45+CD19−CD133+ populations by using either anti-CD133-labeled magnetic beads (Miltenyi Biotec, Auburn, CA) or fluorescent activating cell sorter.

### Coculture with MS-5 and hMSC stromal cells

MCL (bulk and subpopulations) were plated on a pre-established confluent layer of irradiated (25 Gy) MS-5 or hMSC stromal cells as previously described [21]. Once a week, cultures were demipopulated by removing carefully half of the medium from each culture vessel and adding an equal volume of fresh A10 medium. After 4–6 weeks, the unattached population was carefully collected and saved. The monolayer was visually checked to ensure the stromal layer was intact. The CAFC cells were removed from the stromal cell layer by treating the layer with enzyme-free cell disassociation solution (Invitrogen, Carlsbad, CA) followed by two washes in A10 medium. MCL cells were then isolated from the stromal cells using anti-human CD45-conjugated magnetic beads (Miltenyi Biotec, Auburn, CA).

### The effects of different Cell detachment reagents on CD19 and CD133 surface expression

To confirm the lack of CD19 and the expression of CD133 on MCL cells was not due to the enzyme-free PBS-based cell dissociation solution, we examined the effects of different cell detachment reagents on cell surface expression of CD19 and CD133. 3×10^6^ UCB CD5+ B-cells or 5×10^5^ UCB CD133+ cells were incubated for 10 min at 37°C in the indicated cell detachment reagent. Cell detachment reagents (Life Technologies, Rockville, Md.) included Trypsin, Accutase, TrypLE, Collagenase I, Collagenase IV, Dispase II and enzyme-free PBS-based cell dissociation solution. Control cells were incubated in a similar manner in the absence of exogenous enzyme. After incubation, 2 ml of FBS was added to quench enzymatic activity, and cells were washed in A10 medium. Cells were then stained with antibodies for either CD133 (Miltenyi), CD19 (Beckman Coulter) or isotype controls and analyzed by flow cytometry. Four independent experiments were conducted for each reagent and 50,000 events were counted and data plotted as mean fluorescent intensity (MFI).

### CAFC frequency assay

Determination of CAFC frequencies from primary MCL specimens was performed by limiting-dilution analysis in 96-well plates [24], [25], [27]. For each sample, 10-fold serial dilutions of cells (1 to 1×10^5^ cell per well) were prepared and plated in 96-well plates over a pre-established confluent layer of irradiated (25 Gy) MS-5 cells. Twenty-four to thirty-six wells per dilution were plated for each group of cells and maintained at 37°C and 5% CO_2_. Once a week, cultures were demipopulated by removing carefully half of the medium (100 µl) from each well, and adding an equal volume of fresh A10 medium. Wells were evaluated for cobblestone areas weekly for 5–6 weeks with an inverted microscope. Phase contrast dark-cell aggregates consisting of at least 10 small cells defined a cobblestone area (CA). CAFC frequency data analysis was performed using the ELDA website (http:bioinf.wehi.edu.au/software/elsd/index.html) [28].

### *Ex vivo* Self-renewal assay

Cells from 5 week old MCL/MS-5 co-culture flasks were separated into 3 populations of cells 1) bulk unfractionated cells; 2) CD19+CD133− cells and (3) CD19−CD133+ cells by immunomagnetic beads (Miltenyi Biotec, Auburn, CA). Bulk (1×10^5^ cells), CD19+CD133− (1×10^5^ cells) and CD19−CD133+ (1×10^3^ cells) populations were added to fresh, irradiated MS-5 cells and maintained for 5 weeks after which time the number of CA were enumerated. Cells were then fractionated again and the process was repeated 3 times.

### Quiescent cell determination

The presence of quiescent cells was determined by staining the suspension cells and the CAFC with CD45-PC7 for 30 min on ice followed by fixation with 1% paraformaldehyde, then incubated for 15 min at room temperature with permeabilization buffer (BD Bioscience). Cells were then incubated on ice for 45 min with Ki-67-FITC antibody (BD Bioscience) washed twice in PBS containing 2% FBS and resuspended in the same buffer containing 7-AAD.and analyzed by flow cytometry. For the proliferation assay, irradiated MS-5 cells were added to the upper chamber of a 1 µm transwell insert and allowed to adhere overnight. Primary MCL cells depleted of unwanted cells by magnetic beads were added to the upper insert and maintained for 4 weeks for CA formation. At week 5, 10 µM of BrdU was added to both chambers of the transwell plate and incubated for 16 hrs. The CD19+CD133 and CD19−CD133+ MCL subpopulations were processed as described in section “Cell depletion and MCL fractionation”. BrdU incorporation into the CD19+CD133 and CD19−CD133+ subpopulations was determined using the BrdU Cell Proliferation Assay Kit (Cell Signaling). After staining, the cells were analyzed by flow cytometry.

### Drug sensitivity assay

Suspension assay. Bulk (1×10^5^), CD19−CD133+ (1×10^3^), CD19+CD133− (1×10^5^) cells and UCB derived CD133+ (1×10^3^) cells were cultured in the presence or absence bortezomib (15 nM), doxorubicin (50 nM) or fludarabine (15 µg/ml) for 3 days. Cell viability was evaluated by staining with 7-AAD followed by FACS analysis.

Cobblestone Inhibition Assay (CIA). The cell populations described above were cultured for 3 weeks on irradiated MS-5 cells in the presence or absence of bortezomib (15 nM), doxorubicin (50 nM) or fludarabine (15 µg/ml). The number of CAFC forming in the presence of the indicated drug at week 5 was enumerated using an inverted microscope and compared to the number of CAFC forming in the absence of drug. These experiments were repeated three times for each of the 6 patient samples.

### FISH analysis

Cells from patients, CAFC and transplanted mice were evaluated for the presence of the t(11;14) translocation using the dual color dual fusion IgH/CCND1 probe following the manufactures instructions(Abbott Laboratories, Abbott Park, IL, USA). Cells were examined using a triple band-pass filter for Spectrum Green, Spectrum Orange and DAPI. Whenever possible, a minimum of 200 cells were examined.

### Xenotransplantation of unsorted MCL cells into NOD/SCID Mice

Six-week old female NOD/SCID (NOD/Ltsz-Prkdc SCID/J) mice were purchased from Jackson Laboratory (Bar Harbor, ME) and housed in pathogen-free facility at a Rutgers University vivarium. All experimental procedures on the mice were approved by the Institutional Animal Care and Use Committee of the Robert Wood Johnson Medical School. Prior to transplantation, mice were given a sub-lethal dose of radiation (350 cGy) and transplanted. To determine the frequency of MCL-IC, unsorted MCL cells from six patients were mixed with 1×10^6^ irradiated hMSC and injected with cells ranging from 5×10^3^ to 1×10^6^ MCL cells i.p. There were 4 mice per group for each injection dose. Mice were sacrificed when they exhibited signs of illness or up to 20 weeks after injection. Engraftment was determined by flow cytometry by staining cells with a human specific CD45 antibody. The threshold for engraftment was considered to be ≥1% human CD45^+^ cells isolated from the spleen.

### Limiting dilution and serial xenotransplantation of MCL subpopulations

To determine the engraftment potential of the different MCL subpopulations, freshly isolated unsorted, CD19+CD133− MCL cells and CD19−CD133^+^ MCL cells from 4 patients (UPN1, UPN3, UPN5, UPN6) were each mixed with 1×10^6^ irradiated (25 Gy) hMSC, and injected i.p. into NOD/SCiD mice. The MCL cell concentrations injected into mice ranged from 25, to 1×10^6^ cells/mouse. There were 3–6 mice per group for each injection dose. When mice with primary xenografts showed signs of illness, the mice were sacrificed, and the spleen and BM were harvested, pooled and processed for cell fractionation. The unsorted, CD19+CD133− and CD19−CD133+ cells were injected i.p. into secondary recipient NOD/SCID and monitored as described above. Subsequently, secondary tumors were sorted and transplanted into tertiary recipient mice by the same method. Limiting dilution analysis (LDA) was performed using online software provided by WEHI bioinformatics (http:bioinf.wehi.edu.au/software/elsd/index.html) [28].

#### Immunohistochemistry

Tissue samples from tonsils and MCL xenotransplanted mice were fixed in 10% buffered formalin overnight followed by transfer to 70% ethanol. Immunohistochemical staining of the paraffin-embedded tissues was conducted by the Tissue Analytical Services at the Cancer Institute of New Jersey (New Brunswick, NJ). The following antibodies used for tissue staining were purchased from Ventana Medical Systems (Tuscon, AZ): anti-human CD3, CD5, CD19, CD20, CD23, CD45, CD79a, Ki-67, cyclin D1, Pax5 and the appropriate isotype controls.

### Detection of stem cell and B-cell associated markers by qRT-PCR

Total RNA was isolated using the RNAEasy Kit (Qiagen Inc. Valencia, CA, USA) in accordance with the manufacturer's instructions. At least 1×10^6^ cells were harvested from CAFC, suspension cells, and UCB CD5+ B-cells. Only RNA samples with A260/A280 absorbance ratios between values of 1.8–2.0 were used for quantitative real-time polymerase chain reaction (RT-PCR) experiments. Quantitative PCR experiments were carried out with 50–100 ng of RNA using Taqman Universal Master Mix (Applied Biosystems). Human β-actin primers (Applied Biosystems) were used as the endogenous control to normalize expression values of mRNA with the ΔCT method. Nanog (Hs04260366-s1), Pou5f1 (Hs04260367-gH), Ebf1 (Hs00365513-m1), Sox4 (Hs00268388-s1), Bmi1 (Hs00186411-m1), Msi2 (Hs00292670-m1), Pax5 (Hs00277124-m1), CD19 (Hs00174333-m1), CD133 (Hs01009250-m1), Sox11 (Hs00846583-s1), Sox4 (Hs00268388-s1) and Sox2 (Hs01053049-s1) probes and primers were purchased from Applied Biosystems.

### Statistical analysis

Statistical analysis was carried out by the student's t-test or one-way ANOVA as appropriate. A *P*<0.05 was considered to be statistically significant. Kaplan-Meier survival curves and pair-wise comparison of survival curves and determination of *P* values were done using the Log-rank (Mantel-Cox) test. All statistical analysis was conducted using the GraphPad Prism software program (version 5, Graphpad Software).

## Results

### Stromal cells support a putative MCL tumor-initiating cell population

**Figure 1:**
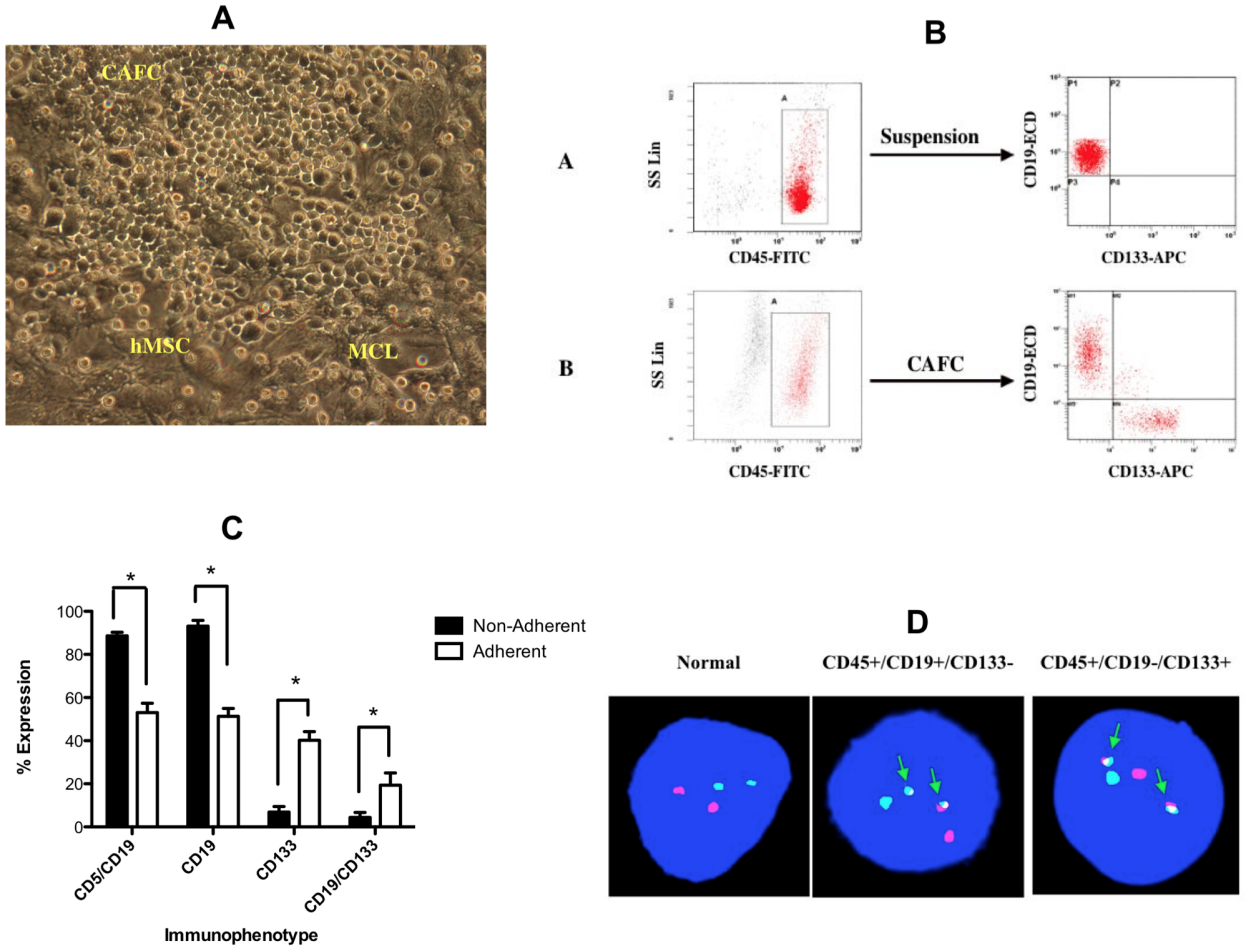
CAFC derived from patients with MCL have a progenitor-like immunophenotype that contains the characteristic t(11;14) translocation. (A) Photograph showing a representative CAFC formed after 5 weeks of co-culture on hMSC. (B) Representative gating and FACS analysis of MCL cells in suspension compared to the CAFC. (C) Immunophenotype analysis of MCL in suspension compared to MCL CAFC (*P<0.001, n = 6 patients). (D) FISH analysis for the t(11;14) translocation in normal, CD19+CD133− and CD19−CD133+ MCL cells.

Upon co-cultivation with MS-5 or hMSC cells, a small population of MCL cells adhered to the stromal layer whereas other cells remained in suspension or weakly attached to the MS-5/hMSC cell layer. Over the next five weeks, the adherent cells formed numerous foci consisting of small round phase-dark lymphoid cells (Fig. 1A). These foci are similar in morphology to CAFCs observed when HSC are cultured with murine or human stromal cells implying that these cells might represent a unique MCL population [23], [27].

To begin to characterize these distinct MCL cell populations, we co-cultured MS-5 and MCL cells and analyzed the suspension and CAFC populations for surface expression of CD45, T-cell (CD3, CD5), B-cell (CD19, CD23) and stem cell markers (CD34, CD133) by flow cytometry. To ensure that the stained cells were not of stromal cell origin, we gated on the cell population that stained positive with anti-human CD45-FITC (Figure 1B). MCL cells from the suspension and adherent foci were positive for CD45, CD38, CD5 and negative for CD3, CD14, CD23 and CD34 surface expression (Table 1). Interestingly, evaluation of CD19 expression on both cell populations revealed that >92% of the cells in suspension expressed CD19 while only 50% of the CAFCs expressed CD19 (Figure 1C). We next evaluated the cells for expression of the stem cell marker CD133. As shown in Figure 1B–C, approximately 40% of the CAFC population expressed the stem cell marker CD133+ while only 1–5% of the non-adherent cells were CD133+.

**Table 1:** Immunophenotype of MCL cells in suspension and CAFC.

Antigen	Suspension (%)	CAFC (%)
CD3	0	0
CD5	99	94^low^
CD14	0	0
CD19	95	20
CD23	0	0
CD34	0	0
CD38	98^mod^	93^low^
CD44	97	98
CD133	3	57

As proof that the CD19−CD133+ cells were derived from the MCL cell population and not from contaminating normal HSC, we evaluated UCB CD5+ B-cells, bulk MCL cells, and the CD19−CD133+ cells for the t(11;14)(q13;q32) translocation that is characteristic of MCL. As shown in Fig. 1D, FISH analysis revealed that both the CD19−CD133+ and the CD19+CD133− cell fractions contained the t(11;14)(q13;q32) translocation, while UCB CD5+ B-cells did not. These data confirm the common genetic origin of MCL-associated CAFC and the bulk MCL population.

These findings prompted us to evaluate fresh samples from MCL patients for CD133+CD19− frequency. In these experiments, MCL cells were depleted of lineage specific (CD2, CD3, CD11b, CD14, CD16 CD34, CD56) cells and stained for CD45, CD19 and CD133 expression. The percentage of CD19−CD133+ cells in the CD45+ population was determined by counting a minimum of 5×10^6^ CD45 + cells by flow cytometry. As shown in Table 2, CD19−CD133+ MCL cells represent 0.11–0.53% of the population. We next determined the CAFC frequency by limiting dilution on irradiated MS-5 stromal cells. As shown in Table 2, the frequency of CAFC in unsorted primary MCL cells ranged from 1/945-1/2308. Finally, the CD133+ cells were sorted and evaluated by FISH for the chromosomal translocation t(11;14). All CD133+ cells from each of the 6 patients contained the t(11;14) translocation signal (Table 2). These data support the hypothesis that the CD19−CD133+ cells are a unique subset of primary MCL cells.

**Table 2:** Percent CD45+CD19−CD133+ cells, CAFC frequency and t(11;14) translocation in unsorted MCL cells.

MCL UPN	CD19−CD133+	CAFC Frequency	FISH t(11;14)
	(%)	(95% CL)	
1	0.11	1/2308	
		(1591–3347)	+
2	0.30	1/1190	
		(851–1665)	+
3	0.14	1/1535	
		(1088–2165)	+
4	0.18	1/1998	
		(1394–2863)	+
5	0.21	1/1377	
		(981–1933)	+
6	0.53	1/945	
		(677–1319)	+

### Cell detachment reagents have no affect on CD19 and CD133 surface expression

Concerned that the enzyme-free, PBS-based cell dissociation solution may adversely affect the cell surface expression of CD19 and/or CD133, we treated UCB CD5+CD19+ and CD133+ cells with 7 commonly used cell dissociation reagents. As shown in Figure S1, none of the reagents demonstrated a significant affect on either CD19 or CD133 expression. Thus the enzyme-free PBS-based cell dissociation solution was used for the remainder of the study.

### CD133+ MCL cells have *ex vivo* self-renewal capacity

**Figure 2:**
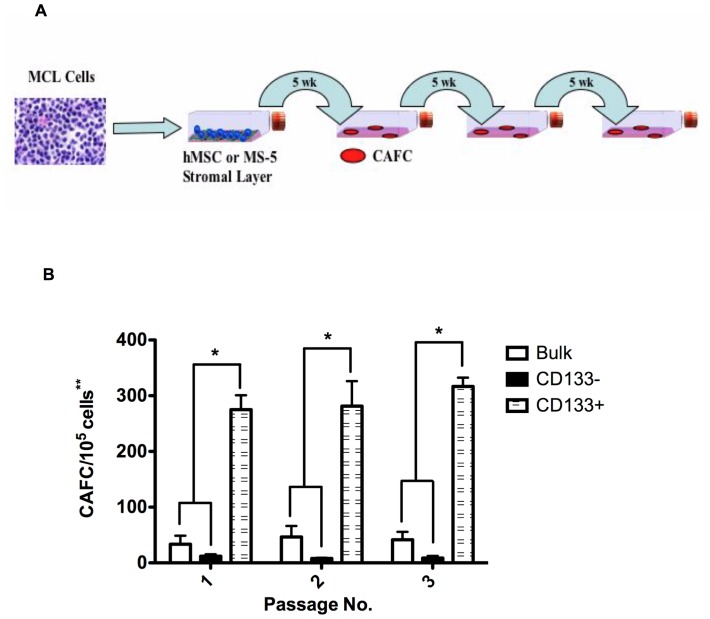
CD19−CD133+ MCL cells demonstrate increased self-renewal, compared to CD19+CD133− MCL cells. (A) Diagram of the self-renewal assay. (B) CAFC analysis comparing bulk, CD133− and CD133+ MCL cells. (*p<0.001, n = 6 patients). ** One hundred times more bulk and CD19+CD133− cells were used compared to the CD19−CD133+ cells.

A main postulate of the cancer stem cells hypothesis is the ability of TICs to self-renew. We evaluated the self-renewal property of the CD19−CD133+ MCL by overlaying a confluent layer of irradiated MS-5 cells with 1×10^5^ MCL cells (bulk or CD133−) or 1×10^3^ CD133+ cells (100-fold fewer cells than used in the bulk and CD133− cultures). After 5 weeks in culture, the cobblestone areas were enumerated. Cells were removed from the flask and sorted into the above subsets and re-plated at the original cell density on fresh cultures of irradiated MS-5 cells (secondary culture; Figure 2A). The procedure was repeated after an additional 5 weeks. As shown in Figure 2B, primary cultures of bulk and CD133− cells formed significantly fewer CAFC compared to CD133+ cells. This difference in CAFC ability was maintained through 3 serial passages. These data demonstrate the self-renewal capacity of the CD133+ MCL cell population *ex vivo*.

### CAFCs have an increased quiescent MCL population compared to the suspension cell population

**Figure 3:**
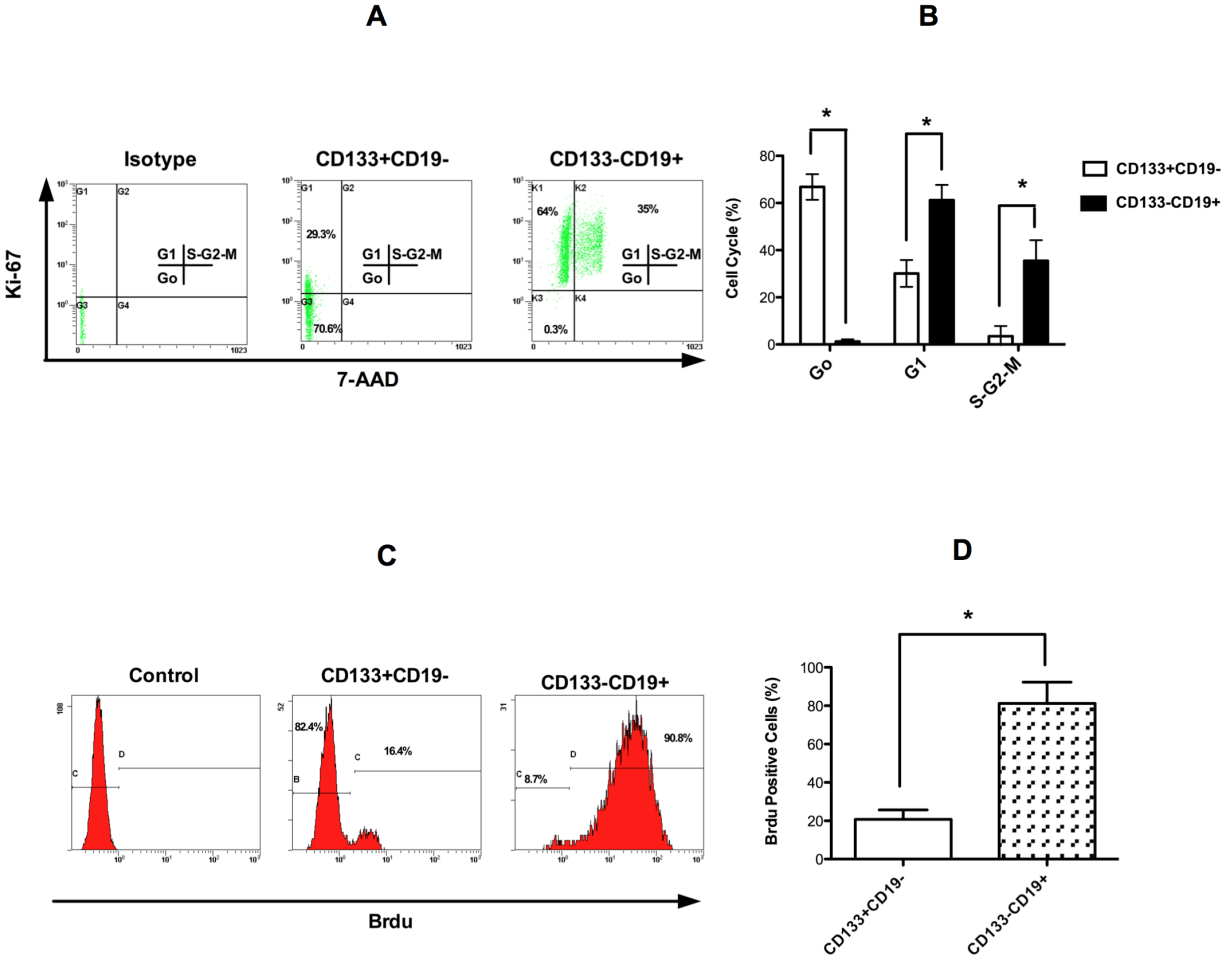
CD19−CD133+ MCL cells demonstrate increased quiescence. (A) A representative dot plot of Ki-67 stained MCL cell subpopulations (UPN1). (B) Bar graph summarizing the data derived from 6 patients (*p<0.01). (C) A representative histogram plot of Brdu incorporated into MCL subpopulations (UPN1). (D) Bar graph summarizing the data derived from 6 patients (*p<0.01).

Because the CAFC demonstrated a primitive immunophenotype and increased self-renewal capacity compared to cells in suspension, we next wanted to determine whether the CD19−CD133+ cells have a decreased proliferation rate compared to the CD19+CD133− MCL cells. In this series of experiments proliferation was evaluated by Ki-67/7-AAD staining and BrdU incorporation. As shown in Figure 3A–B, the CD19−CD133+ cell population had a significantly greater number of cells in Go (73%) compared to the CD19+CD133− (2%) population (p<0.01) as demonstrated by the reduced labeling with the nuclear antigen Ki-67. In contrast, the CD19+CD133− cells had significantly more cells in G1 and S-G2-M (64% and 35%) compared to the CAFC population, 27% and 8% respectively (p<0.01). As additional evidence of the quiescent nature of the CD19−CD133+ cells, BrdU incorporation was significantly reduced compared to the CD19+CD133− subpopulation (Figure 3C–D).

### CD19−CD133+ MCL cells exhibit increased drug resistance

**Figure 4:**
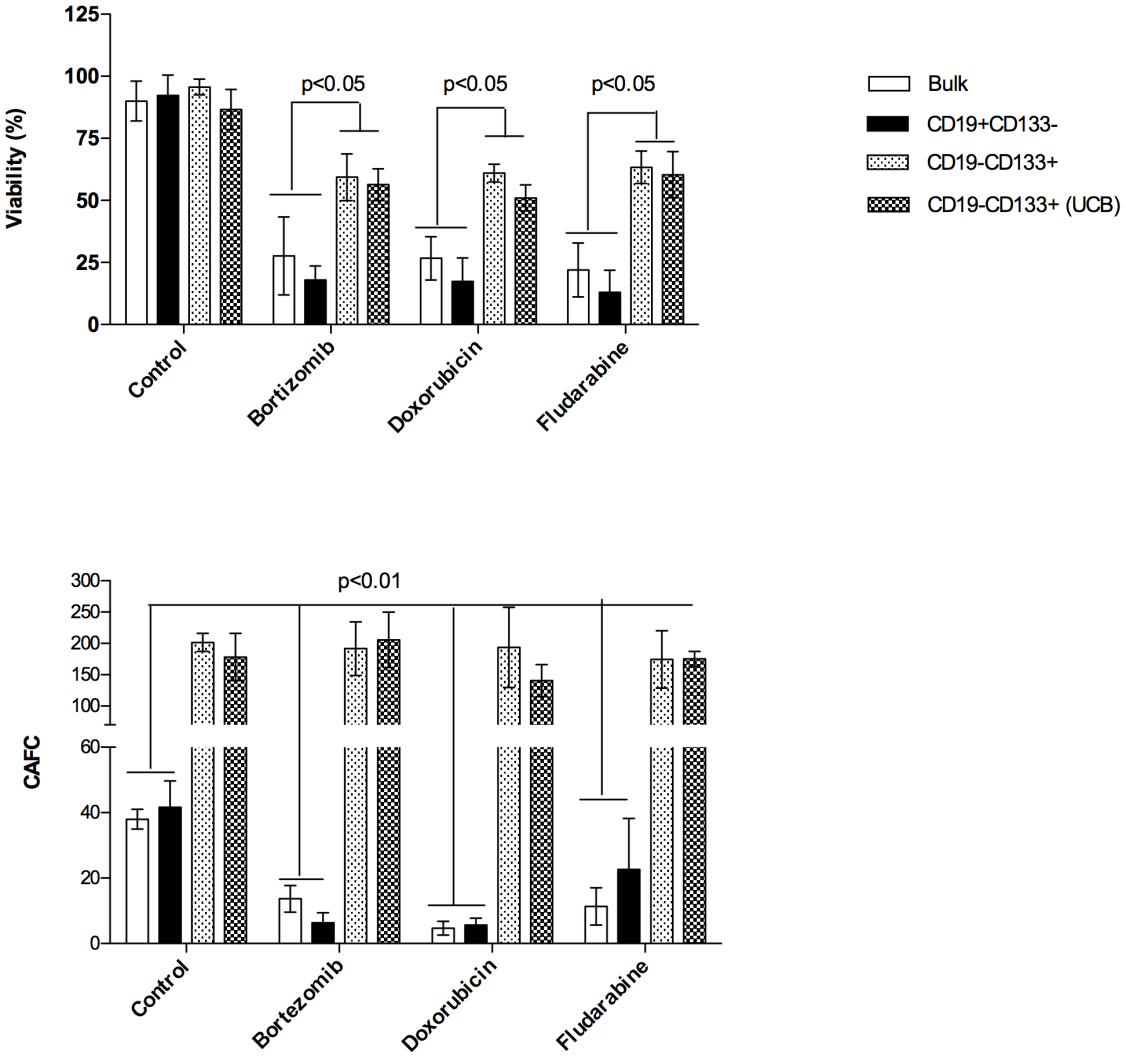
CD19−CD133+ MCL cells demonstrate increased drug resistance. (D) Sensitivity of different subpopulations of MCL to Bortezomib (15 nM), doxorubicin (50 nM) and fludarabine (15 µg/ml) was evaluated in suspension assay and CAFC assay. (A) MCL subpopulations were cultured alone in the presence or absence of drug for 48 hrs then evaluated for cell viability by 7-AAD staining and FACS analysis *(p<0.01, n = 6 patients). (B) MCL subpopulations were cocultured with MS-5 cells in the presence or absence of drug. The number of CA was determined at week 5 *(p<0.001, n = 6 patients).

Several studies have shown that CSC/TICs are more resistant to chemotherapy, and that chemotherapy can selectively enrich for TICs both *in vitro* and *in vivo* [14], [16]. To asses the relative efficacy of bortezomib, doxorubicin and fludarabine on bulk MCL cells and MCL subpopulations, cells from 6 patients were incubated with the indicated drug concentration. After 72-hour exposure in suspension culture the cultures were evaluated for cell viability. Figure 4A shows that CD19−CD133+ MCL and CD133+ UCB cells exhibit significantly (p<0.05) increased drug resistance compared to bulk and CD19+CD133− MCL cells.

We next examined the effects of bortezomib, doxorubicin and fludarabine on CAFC formation. As shown in Figure 4B, UCB CD133+ cells and CD19−CD133+ MCL cells treated with either bortezomib, doxorubicin or fludarabine demonstrated no significant difference in CAFC formation compared to untreated UCB and MCL CD133+ control cells. In contrast, bulk and CD19+CD133− MCL populations demonstrated a significant decease (p<0.01) in CAFC formation compared to untreated bulk and CD19+CD133− MCL cells. Taken together, this data demonstrates that the CD19−CD133+ MCL cells exhibit greater drug resistance than either the bulk or CD19+CD133− MCL cells.

### Stem cell and B-cell marker expression in CD133+ and CD133− MCL cells

**Figure 5:**
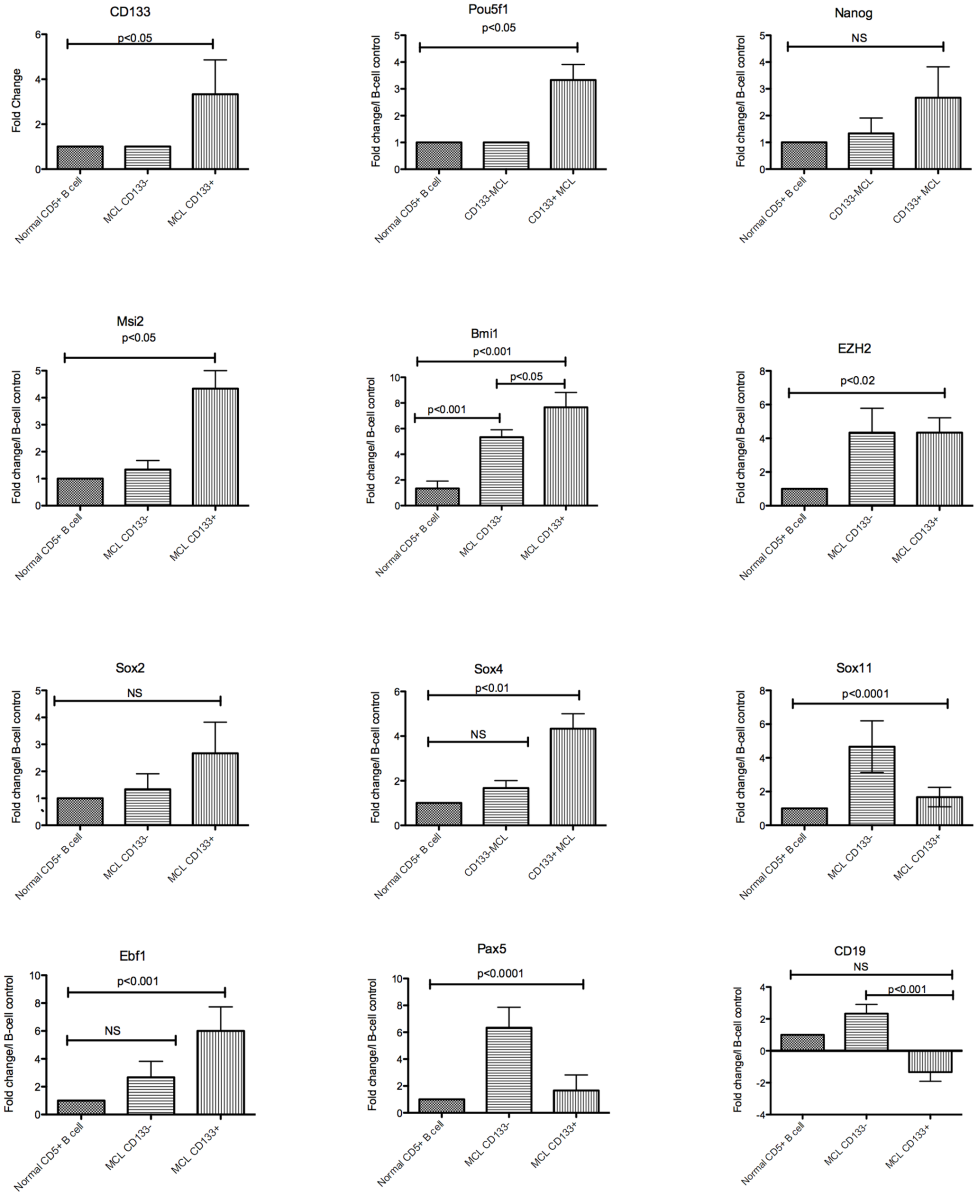
Expression of of self-renewal and B-cell differentiation associated transcription factors. qRT-PCR was conducted on RNA isolated from UCB CD5+ B-cells, CD19+CD133− and CD19−CD133+ subsets of MCL cells.

The above data demonstrate the in vitro self-renewal capacity of CAFC-derived CD19−CD133+ MCL cells. We next wanted to determine the extent to which the CD19−CD133+ cells expressed any of the following stem cell-associated markers: CD133, Pou5f1, Nanog, Msi2, Bmi1 and Sox2. In this series of experiments, we conducted qRT-PCR on RNA isolated from CD19−CD133+, CD19+CD133− MCL cells. We used UCB CD5+ B-cells as controls. As shown in Figure 5, CD19−CD133+ MCL cells expressed significant levels of CD133, Pou5f1 and Msi2 compared to UCB CD5+B-cells and CD133− MCL cells. Expression levels of Bmi1 were significantly higher in the CD133+ and CD133− MCL compared to CD5+ B-cells. Not surprisingly, Bmi1 expression in the CD133+ cells was also significantly higher than the CD133− MCL cells. In addition, CD133+ MCL cells showed a trend towards increased levels of Sox2, and nanog expression compared to CD133− MCL cells and UCB CD5+ B-cells but did not reach statistical significance.

Several reports have shown that most MCL cells express high levels of Sox11 and in some cases low/moderate expression of Sox4, a known regulator of B-cell development [29]–[34]. It has also been reported that some functions of Sox genes may functionally substitute for one another [33]. Due to the lack of significant expression of Sox2, we decided to compare Sox4 and Sox 11 expression. As shown in Figure 5, CD133+ MCL expressed 4-fold higher (p<0.01) Sox4 compared to CD133−MCL cells and UCB CD5+ B-cells. We next evaluated Sox 11 expression (Figure 5) and show that Sox 11 was ∼5-fold higher (p<0.0001) compared to CD133+ MCL cells and UCB CD5+ B-cells.

We next evaluated the expression levels of the B-cell associated transcription factors, Ebf1, Pax5 and the Pax5-regulated gene CD19. Ebf1 RNA was 6-fold higher (p<0.001) in CD133+ MCL than CD133− MCL cells and UCB CD5+ B-cells. In contrast, Pax 5 RNA expression was 6-fold higher (p<0.0001) in CD19+CD133− MCL cells compared to CD133+ MCL cells and UCB CD5+ B-cells. Finally, CD19 RNA expression in UCB CD5+ B-cells and CD133− MCL cells were ∼6-fold higher (p<0.001) than CD133+ MCL cell population.

### Engraftment of primary MCL cells in NOD/SCID mice require human mesenchymal stromal cells

In preliminary xenotransplantation experiments, injection (i.v. or i.p.) of 1×10^7^ unsorted MCL cells into irradiated NOD/SCID mice failed to demonstrate engraftment after 20 weeks as assessed by hCD45 expression (data not shown). The inability to initiate MCL tumors from any of the patient samples may be attributed to (i) the lack of MCL-ICs in the population or (ii) the inability of human MCL cells to respond to murine microenvironmental signals. Based on the ability of hMSC and murine MS-5 cells to support CAFC formation of MCL cells, and recent studies demonstrating the ability of bone marrow stromal cells to increase the in vitro survival of primary MCL cells [35], [36] we investigated the ability of MS-5 and hMSC to support MCL cell xenotransplantation in NOD/SCID mice.

In this experiment, irradiated mice were injected i.p. with 1×10^6^ MCL cells alone or co-injected with 1×10^6^ irradiated MS-5 or hMSC. As shown in Figure S2 all mice co-injected with MCL and hMSC became moribund between 100–150 days and had significant (p = 0.001) engraftment of human CD45 cells in the spleen (Figure S3) compared to mice injected with MCL cells alone or co-injected with MS-5 cells. Even after 200 days post injection, mice injected with MCL cells alone or with MS-5 cells remained healthy and disease free and showed no evidence of engraftment. On necropsy of the mice, we observed differences in peritoneal cavity fat tissue. Control mice not injected with cells had minimal fat tissue, while mice injected with MCL/hMSC cells had large white tumor mass in the peritoneal cavity. In contrast, mice injected with the MS-5 cells presented with a small mass of yellow fat tissue (data not shown). Based on these data MCL/hMSC co-injection was used in all remaining xenotransplantation assays.

### Tumor-initiating capacity of unsorted MCL cells in NOD/SCID mice

To determine the tumor-initiating capacity of primary MCL, we injected various amounts of unsorted MCL cells from six patients into NOD/SCID mice. As shown in Table 3 and Figure S4) all mice injected with the highest number of MCL cells (1×10^6^/mouse) demonstrated engraftment. Using quantitative limiting dilution analysis, we found that MCL-IC frequency ranged from 1/109,742 (UPN1) to 1/9,649 (UPN6) (Table 3). We next calculated the average MCL-IC frequency for the six patient samples by combining the engraftment data from Table 3 followed by LDA. This analysis showed that on average there was one MCL-IC per 4.4×10^4^ cells (95% confidence interval: 1/60,402 to 1/31,538), thus demonstrating that only a small subset of MCL cells have the capacity to initiate MCL growth.

**Table 3:** Xenograft Limiting dilution assays of unfractionated primary MCL-IC in NOD.SCID mice.

Patient	Cells injected	No. of mice injected	No. of mice with tumors	MCL-IC frequency-1		
				Lower	Estimate	Upper
	1×10^6^	4	4			
	5×10^5^	4	4			
UPN1	1×10^5^	4	3	261038	109742	46136
	5×10^4^	4	1			
	1×10^4^	4	0			
	5×10^3^	4	0			
	1×10^6^	4	4			
	5×10^5^	4	4			
UPN2	1×10^5^	4	4	41916	17620	7407
	5×10^4^	4	4			
	5×10^4^	4	2			
	1×10^4^	4	0			
	1×10^6^	4	4			
	5×10^5^	4	4			
UPN3	1×10^5^	4	4	180541	77457	33231
	5×10^4^	4	1			
	1×10^4^	4	0			
	5×10^3^	4	0			
	1×10^6^	4	4			
	5×10^5^	4	4			
UPN4	1×10^5^	4	3	336121	142456	60376
	5×10^4^	4	0			
	1×10^4^	4	0			
	5×10^3^	4	0			
	1×10^6^	4	4			
	5×10^5^	4	4			
UPN5	1×10^5^	4	4	76763	34664	15653
	5×10^4^	4	3			
	1×10^4^	4	1			
	5×10^3^	4	0			
	1×10^6^	4	4			
	5×10^5^	4	4			
UPN6	1×10^5^	4	4	24150	9649	3855
	5×10^4^	4	4			
	1×10^4^	4	3			
	5×10^3^	4	1			
	1×10^6^	24	24			
	5×10^5^	24	24			
Total	1×10^5^	24	22	60402	43646	31538
	5×10^4^	24	16			
	1×10^4^	24	6			
	5×10^3^	24	1			

### Tumor-initiating capacity of sorted MCL cells in NOD/SCID mice

**Figure 6:**
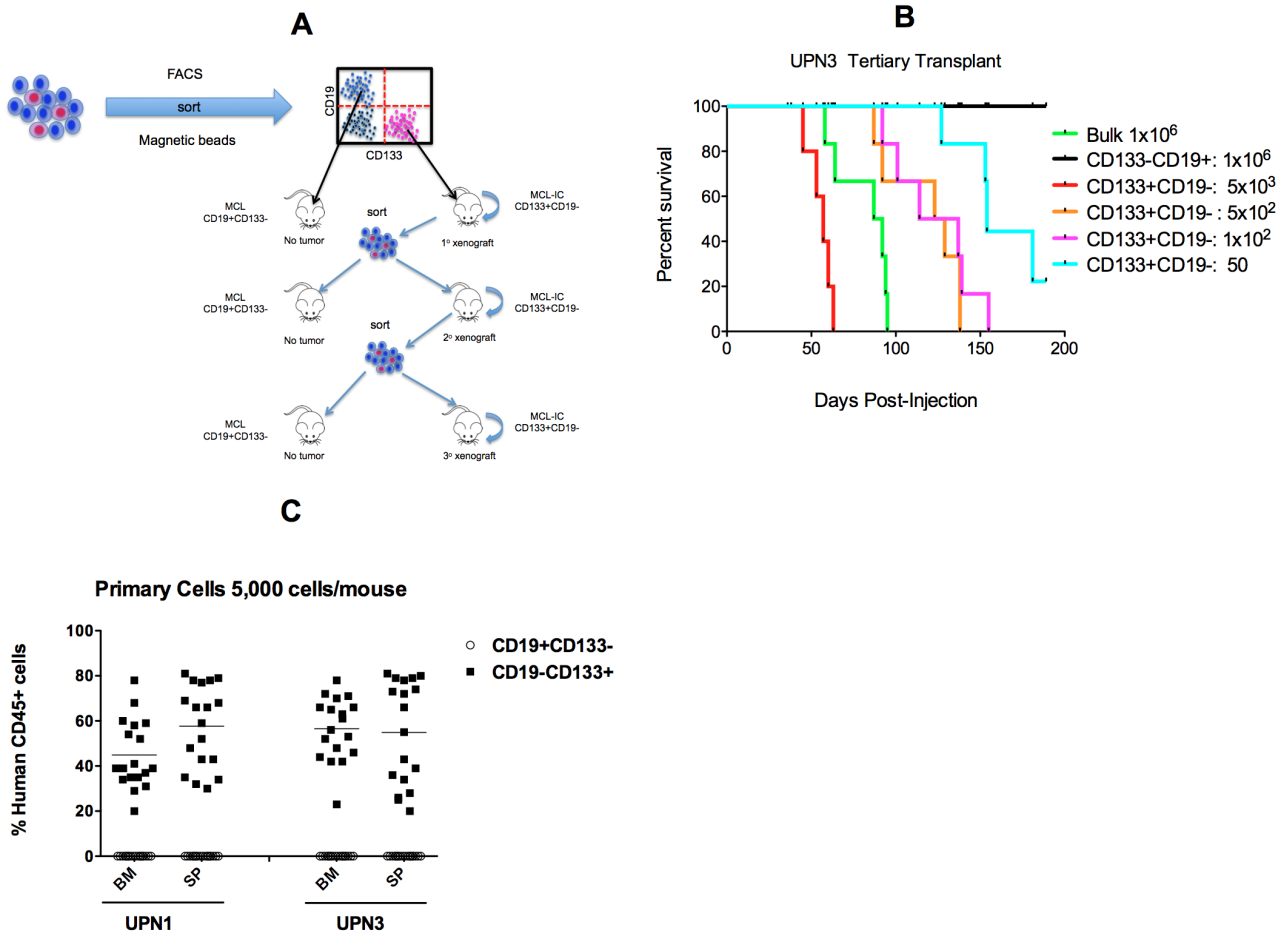
Only CD19−CD133+ MCL engraft NOD/SCID mice and recapitulate the disease. (A) Diagram of serial transplantation scheme. (B) Representative survival curve of NOD/SCID mice injected with the various MCL subpopulations (UPN3). (C) Representative hCD45 engraftment profile of bone marrow (BM) and spleen (SP) from mice injected with either CD19+CD133− or CD19−CD133+ subpopulation (5,000 cells/mouse).

Functional characterization via quantitative xenotransplantation assay using immunodeficient mice is considered the “gold-standard” for TIC indentification [37]. Thus, in this series of experiments, we examined the ability of MCL subpopulations to engraft NOD/SCID mice (Figure 6). Cells from 4 patients (UPN1, UPN3, UPN5, UPN6) were sorted into CD19+CD133− and CD19−CD133+ subpopulations. These two subpopulations, as well as the bulk MCL cells, were tested for their ability to engraft NOD/SCID mice. Due to limited availability of cells, we were unable to evaluate UPN2 and UPN4. As shown in Table 4, inoculation of 10^6^ bulk cells from each of the four patients resulted in engraftment of NOD/SCID mice. In mice injected with sorted populations, engraftment was only observed using CD19−CD133+ cells (Table 4 and Figure S5). In contrast, there was no detectable engraftment with the CD19+CD133− subfraction despite injecting 200-fold more cells than used for the CD19−CD133+ cells

**Table 4:** Limiting dilution and serial transplantation analysis of Primary MCL-IC.

Patient	Cell subtype	Cells injected	1° Transplant	2° Transplant	3° Transplant	**MCL-IC
						Frequency^−1^
						(95% CI)
	Bulk	1,000,000	6/6	*ND	ND	
	CD133−CD19+	1,000,000	0/6	0/6	0/6	690
UPN1	CD133+CD19−	5000	6/6	6/6	6/6	(271–1753)
	CD133+CD19−	500	ND	ND	4/6	
	CD133+CD19−	100	0/6	0/6	0/6	
	CD133+CD19−	50	ND	ND	0/6	
	Bulk	1,000,000	5/6	ND	ND	
	CD133−CD19+	1,000,000	0/6	0/6	0/6	
UPN3	CD133+CD19−	5000	6/6	5/6	6/6	435
	CD133+CD19−	500	ND	ND	4/6	(189–9988)
	CD133+CD19−	100	ND	ND	2/6	
	CD133+CD19−	50	ND	ND	0/6	
	Bulk	1,000,000	4/4	4/4	4/4	
	CD133−CD19+	1,000,000	0/6	0/6	0/6	
UPN5	CD133+CD19−	5000	3/3	3/3	3/3	149
	CD133+CD19−	500	1/3	2/3	3/3	(70–319)
	CD133+CD19−	100	0/6	0/6	1/6	
	CD133+CD19−	50	0/6	0/6	0/6	
	CD133+CD19−	25	0/6	0/6	0/6	
	Bulk	1,000,000	3/3	3/3	3/3	
	CD133−CD19+	1,000,000	0/6	0/6	0/6	
UPN6	CD133+CD19−	5000	3/3	3/3	3/3	47
	CD133+CD19−	500	0/3	3/3	3/3	(25–89)
	CD133+CD19−	100	0/6	4/6	6/6	
	CD133+CD19−	50	0/6	1/6	4/6	
	CD133+CD19−	25	0/6	0/6	1/6	
	Bulk	1,000,000				
	CD133−CD19+	1,000,000				
Total	CD133+CD19−	5000			18/18	279
	CD133+CD19−	500	ND	ND	14/18	(187–418)
	CD133+CD19−	100			9/24	
	CD133+CD19−	50			4/24	
	CD133+CD19−	25			1/12	

* ND = Not done.

**MCL-IC frequency was calculated using the 3° transplant data.

An important measure of stem cell function is the self-renewal capacity as demonstrated by serial transplantation in immunocompromised mice [13]. We found that only the CD19−CD133+ subpopulation was capable of secondary and tertiary engraftment, indicating the long-term self-renewal potential of MCL-IC (Table 4 and Figure S5). As shown in Figure 6B–C and Table 4, the frequency of MCL-IC was patient sample dependent and ranged from 1/47 to 1/690. The average frequency for the sorted CD19−CD133+ was 1/279 and represents a 156-fold increase in MCL-IC enrichment when compared to the average frequency in the unsorted cells (1/43,646; Table 3). Immunophenotypic and FISH analysis of engrafted hCD45+ cells revealed that the engrafted cells had the same characteristic immunophenotye as the patients at diagnosis (CD5+CD19−CD23−) and contained the t(11;14) translocation consistent with MCL.

Several reports have shown that sequential passage of TICs in immunocompromised mice resulted in an increased frequency of TICs [38], [39]. Based on these reports we compared the MCL-IC frequency in primary, secondary and tertiary xenografts. A shown in Figure S5B, with each passage in mice there was a small but significant increase in MCL-IC frequency. The reason for the observed increase in MCL-IC frequency is unknown but may be due to adaptation of the cells to the murine microenvironment and warrants additional research.

### MCL CAFC contain MCL-ICs capable of in vivo self-renewal capacity in NOD/SCID mice

We next investigated the ability of CAFC derived CD19−CD133+ to be serially transplanted at limiting dilutions into NOD/SCID mice. CAFC derived from patients UPN1 and UPN5 were fractionated into CD19+CD133− and CD19−CD133+ populations and co-injected with hMSCs as above. As shown in Table 5, only the CAFC derived CD19−CD133+ cells were capable of serially engrafting NOD/SCID mice. The frequency of CD19−CD133+ MCL-IC was 1/65 and 1/44 for UPN1 and UPN5 respectively. As few as 25 CD19−CD133+ cells/mouse cells were able to induce tumors, thus confirming that the CD19−CD133+ CAFC contain stem/progenitor-like activity.

**Table 5:** Limiting dilution and serial transplantation analysis of CAFC derived MCL-IC.

Patient	Cell Type	Cells Injected	1° Transplant	2° Transplant	3° Transplant	TIC Frequency^−1^
						(95% CI)
	CD133−CD19+	5,000,000	0/3	ND	ND	
	CD133+CD19−	5000	4/4	4/4	4/4	
UPN1	CD133+CD19−	500	4/4	4/4	4/4	65
CAFC	CD133+CD19−	100	3/4	4/4	4/4	(43–98)
	CD133+CD19−	50	2/6	3/6	4/6	
	CD133+CD19−	25	1/6	1/6	3/6	
	CD133−CD19+	5,000,000	0/3	ND	ND	
	CD133+CD19−	5000	4/4	4/4	4/4	
UPN5	CD133+CD19−	500	4/4	4/4	4/4	44
CAFC	CD133+CD19−	100	3/6	5/6	5/6	(33–63)
	CD133+CD19−	50	3/6	5/6	5/6	
	CD133+CD19−	25	2/6	2/6	3/6	

### Necropsy and histopathological analysis

Necropsy and histopathological analysis was performed on control mice and mice transplanted with the different MCL subpopulations. As shown in Figure S6A, mice injected with CD19−CD133+ cells often presented with swollen abdomens while control mice and mice injected with CD19+CD133−cells exhibited no such swelling. On gross necropsy, CD19−CD133+ mice often contained a large peritoneal tumor mass (Fig. S6B) and an enlarged spleen (Fig. S6C) compared to control mice. As shown in Figure 5C, analysis of tumor, bone marrow (BM) and spleen (SP) from sacrificed mice revealed that only the mice injected with the CD19−CD133+ fraction had engraftment of the injected cells as determined by hCD45 staining.

We next evaluated tissue from tumor bearing mice (tumor mass, spleen, intestine and kidney) by immunohistochemical analysis for the expression of CD3, CD5, CD19, CD20, CD23, CD45, CD79a, Ki-67, cyclin D1, and Pax5. Control tissue (tonsil) stained positive for all antigens examined with cyclin D1 weakly expressed and were negative for the t(11;14) translocation (Figures S7, S8, S9). In contrast, lymphoma cells in the tumor mass, spleen, intestine and kidney were positive for all antigens examined except CD3 and CD23. In addition, lymphoma cells were strongly positive for cyclin D1 and contained the t(11;14).

## Discussion

The CSC hypothesis suggests that only a small subset of cells within a tumor has the capacity to initiate and sustain tumor growth. If true, this model would provide a foundation for the development of more efficacious lymphoma therapies by targeting tumorigenic TICs. It is believed that TICs share common traits with their normal stem cell counterparts, such as their ability to self-renew, express a primitive immunophenotype, resistance to clinically used drugs and the ability to recapitulate tumor heterogeneity [40]–[42].

Several recent studies suggest the existence of TICs in MCL [7], [9], [43]. The first by Brennan et al [43] used ALDH expression to identify a clonogenic population in MCL cell lines and four primary samples. They reported that in the 4 primary samples the ALDH+ cells ranged from 0.40 to 7.85% of the population. Unfortunately, the authors did not characterize the clonogenic population for additional stem cell markers such as CD34 and CD133 or perform limiting dilution analysis or serial transplantation in immunocompromised mice. The second study by Chen et al [9] isolated potential MCL-IC based on the lack of CD19 expression and injecting the fractions into NOD/SCID mice and demonstrated serial engraftment of the CD19− population only. They also evaluated other potential MCL-IC markers including the HSC marker CD34 but not CD133 and concluded that only the absence of CD19− expression fractionated the MCL-IC cells. More recently, Teshima et al, identified side population (SP) cells from MCL cell lines and patient samples and demonstrated that the SP cell population had self-renewing and tumor-initiating characteristics [7]. Interestingly, they also demonstrate that deregulation of Bmi1 and microRNA-16 collaborated to enhance the anti-apoptotic activity of the SP population. Although these data are consistent with TIC-like activity, the investigators did not characterize the immunophenotype of the SP cells or conduct the serial limiting-dilution transplantation assay that is considered the “gold standard” for TIC.

To gain further insight into the existence of MCL-IC we utilized an *ex vivo* co-culture similar to the previously developed CAFC assay used to study normal HSC and progenitor cells [23], [25], [44]–[47]. Using this system we demonstrate for the first time that human mesenchymal stromal or the murine BM stromal cell line MS-5 can be used to prospectively identify and expand a unique subpopulation of primary MCL. More specifically, during long-term co-culture we observed clusters of phase-dark cells beneath the stromal layer. These clusters are morphologically similar to CAFC that form under stromal cell monolayer when normal HSC are co-cultured with BM stromal cells [25], [44], [46]. In this study, immunophenotypic characterization of MCL associated CAFCs reveal that these clusters are composed of cells with a unique and primitive phenotype (CD45+CD19−CD133+) compared to cells that remain in suspension and the bulk population (CD45+CD19+CD133−). This immunophenotype is similar to the recently described phenotype for leukemia-initiating cells associated with childhood B-ALL [48] and is in partial agreement with the study of MCL CD19− cells reported by Chen et al [9]. To rule out the possibility that the CAFC formation was due to contaminating normal HSC, FISH analysis was performed on sorted CD45+CD19−CD133+, CD45+CD19+CD133− (bulk MCL cells) and UCB CD5+ B-cells. Only the CD45+CD19−CD133+ and bulk MCL populations contained the t(11;14) translocation supporting our hypothesis that the CAFC were of MCL origin. The CSC hypothesis also proposes that this compartment should exhibit stem cell properties of self-renewal, quiescence, and drug resistance. Consistent with these tenets, our *ex vivo* self-renewal assay established that only the CD19−CD133+ subpopulation of CAFC have the capacity to be serially passage and reform CAFC on either hMSC or MS-5 stromal cells. Furthermore, cell cycle and cell proliferation experiments revealed that the majority of the CD19−CD133+ cells are in Go and incorporate relatively little BrdU compared to the bulk MCL cells. We also demonstrate that the MCL-IC express self-renewal transcription factors (Pou5f1 and Msi2), polycomb proteins (Bmi1 and EzH2) and the B-cell associated transcription factors Sox4 and Ebf1. More importantly, we found that the putative MCL-IC cells were relatively resistant to several agents (bortezomib, doxorubicin or fludarabine) with distinct mechanisms of action. Moreover, this resistant phenotype was observed in suspension cultures as well as in co-culture with hMSC. Taken together, these data are in strong agreement with the predictions of the CSC hypothesis and support the hypothesis that the CD19−CD133+ cells represent MCL-IC.

The SRY (sex determining region Y)-box family of transcriptions factors play an important role in survival, proliferation and differentiation in numerous processes in embryonic stem cells, adult stem cells and TICs [33], [49]. MCL cells have been reported to over-express Sox 11, and to a lesser extent Sox4, a known regulator of B-cell development [31]. We found that Sox4 is over expressed in MCL-IC but not the bulk or UCB CD5+ B-cells. This observation is consistent with the fact that Sox4 is required for B-cell differentiation, as Sox4-null hematopoietic cells transplanted into wild-type mice remain blocked at the pro-B cell stage [49], [50]. More recently, Sox11 has been shown to be overexpressed in aggressive forms of MCL and is speculated to act as an oncogene [29], [30], [51], [52]. In contrast to Sox 4 expression, Sox11 is highly expressed in the bulk (CD19+CD133−) and not expressed in the MCL-IC or in UCB CD5+ B-cells. The switch from Sox4 expression in MCL-IC to Sox11 expression in the mature bulk MCL cells is surprising. The molecular and biological consequence of the switch from Sox4 to Sox11 expression will require additional research.

Martinez-Climentet et al, hypothesized that MCL stem cells, if they exist, are most likely derived from the common lymphoid progenitors (CLP) or B-cell progenitors [53]. The immunophenotype and transcription factor profile of our putative MCL-IC are in line with their hypothesis [53]. Based on the immunopheotype and transcription factor profile, we speculate MCL-IC are derived from pro-B-cells. This is supported by the primitive immunophenotype of the MCL-IC (CD133+, CD38^low^, CD19−), expression of transcription factors known to be involved in ESC and HSC self-renewal, along with the expression of the B-cell regulatory transcription factors Sox4, Ebf1 and Pax 5.

Our studies show that the CD19−CD133+ MCL population exhibits features consistent with the CSC hypothesis. To more thoroughly evaluate the stem cell properties of the CD19−CD133+ subpopulation we utilized the serial transplantation assay in immunodeficient mice, which is considered the gold standard for identifying normal and cancer stem cells [54]. Interestingly, in a preliminary xenotransplantation assays injection of bulk MCL cells or bulk cells coinjected with irradiated murine MS-5 cells were unsuccessful in producing tumors in NOD/SCID mice maintained for up to 20 weeks. Due to the fact that NOD/SCID mice form spontaneous tumors the experiment was terminated at 20 weeks. In light of their ability to support MCL CAFC formation, the lack of engraftment with MCL cells coinjected with murine MS-5 cells was originally surprising. This discrepancy may be explained by several reports showing that MS-5 cells only differentiate into adipocytes, and are capable of supporting human HSC growth and survival, but not their differentiation into lymphoid cells [55]–[57]. In the first study, Brouard et al reported that only MS-5 cells expressing human IL-3 were able to support the engraftment of human stem cells in mice [55]. In a second study, Hubin et al demonstrated that MS-5 cells injected alone into mice rapidly differentiate into adipocytes and were unable to support hematopoiesis [56]. They also demonstrated that MS-5 cells when co-injected with hematopoietic cells into the kidney capsule were only able to regulate hematopoietic stem cell differentiation into cells of the myeloid lineage but not lymphoid [56]. Thirdly, it has also been demonstrated by in vitro experiments that MS-5 cells can only be induced to differentiate into adipose tissue and not into osteogenic or chondrocytes [56] These reports are consistent with our preliminary data in which mice injected with only MS-5 cells or co-injected with MCL cells contained small yellow peritoneal mass consisting of only adipocytes. Adipocytes have also been reported to inhibit B-cell development [58], [59]. Thus the differentiation of MS-5 cells into adipocytes may have prevented the development and engraftment of MCL cells.

The lack of engraftment of bulk MCL alone or coinjected with MS-5 cells into mice suggest that the murine microenvironment may be sub-optimal for the survival and growth of primary MCL-IC. This is based on (i) multiple reports demonstrating that engraftment of normal and CSC cells are improved by the addition of human cytokines and/or human accesory cells in immunodeficient mice [35], [54], [60], [61], and (ii) we originally identified this population of MCL cells by their the ability to form CAFC on hMSC. To test this possability, we injected (i.p.) mice with 1×10^6^ unsorted MCL cells with or without irradiated hMSCs. Mice coinjection with bulk MCL cells and irradiated hMSC demostrated efficient engraftment with cells from each of the 6 patient samples (Figure S1 and S2). In contrast no engraftment was obseverd in the absence of hMSC support. Using the limiting dilution assay we calculated the frequency of the CD19−CD133+ MCL TIC subset to range from 1/109,038 to 1/9,649 (Table 2). By combining the data from the 6 patients we calculated the average frequency to be 1/43,646. This frequency is consistent with TIC frequencys reported for other cancer types [13], [62]–[65].

In this study we have identified and partially characterized MCL-IC from primary patient samples on the basis of their ability to form CA on a stromal layer. Cells comprising the CAFC were characterized by the expression of the stem cell marker CD133, CD5low, CD38low and lack of the pan B-cell marker CD19. Utilizing the limiting dilution assay combined with serial transplantation we demonstrate that only the CD19−CD133+ subset was proficient at initiating human MCL after transplantation into NOD/SCID mice co-injected with hMSC. We also show that the MCL-ICs were capable of establishing MCL in secondary and tertiary mice. Mice transplanted with as few as 25 MCL-IC cells per mouse proliferated and differentiated into lymphoma that were phenotypicaly similar to the patients original tumor.

Chen et al, reported the identification of MCL-IC cells with an immunophenoytpe of CD45+CD19− that contained the t(11;14) translocation and was the only population of MCL cells with the ability to engraft NOD/SCID mice [9]. Samples in that study were obtained from patients with stage 4 disease by apheresis, and the population studied represented 8–15% of the MCL cells. CD133 staining was not reported in that study [9]. In contrast, using our complementary *ex vivo* and *in vivo* systems we have identified a more primitive subpopulation of MCL-IC (CD45+CD19−CD133+) and demonstrate that this population represents <1% of the bulk disease. We speculate that the differences in MCL-IC frequency may be due to stage of disease, sample collection (veinipuncture vs apheresis) and/or other differences in sample procurement and analysis.

Studies by Hielscher et al. in an EBV infected MCL cell line (Granta 519) studied SP cells and subpopulations that express CD133/CD44 [66]. They also studied the impact of matrigel on colony and tumor formation in these populations. Those studies did not evaluate the CD133+/CD44+ or CD133−/CD44− cells for their capacity to retain Hoechst 33342 or establish the immunophenotype of the SP cells. The presence of CD133+ cells in the cell line is interesting, however there were no limiting dilution or serial transplantation assays demonstarting the biological characteristics of these cells. Furthermore, the relevance of these studies to our work is questioned in studies by Daibata et al. using isogenic EBV− and EBV+ MCL cell lines to demonstrate that only the EBV+ cells possessed malignant phenotypes, such as growth in low serum, colony formation in soft agar, and tumor formation in nude mice [67]. These data suggest that EBV+ MCL cell lines may exhibit unique biological properties that differ from both EBV− MCL cell lines and primary MCL cells. Therefore, the relevance of studies using EBV transformed cell line(s) to our studies of primary MCL cell colony formation and tumorigenesis is unknown.

Our findings have several important implications. First, we demonstrate for the first time that the classic CAFC assay can be used as a functional assay for the prospective identification and expansion of MCL-TICs. This is in contrast to the more conventional methods used to identify CSC/TICs such as surface marker expression or isolation as side population cells. Since our assay is based on the ability of the putative TIC to form CAFC and not on immunophenotype this assay may be applicable to the identification and expansion of TICs in other hematological malignancies where the immunophenotype of the putative TIC population is unknown. More importantly, the use of the CAFC assay to identify and quantitate MCL-IC at diagnosis would offer the opportunity to study this cell population during treatment. Furthermore assays of drug sensitivity based upon CAFC inhibition might identify new agents specifically targeting the CD19−CD133+ cells. Finally, the establishment and manipulation of these new and complementary MCL models will provide insights into the molecular, genetic and microenvironmental mechanisms underlying MCL pathogenesis and may lead to the identification of novel treatment strategies.

## Supporting Information

Figure S1 **The effects of different Cell detachment reagents on CD19 and CD133 surface expression.** CD19+ and CD133+, cells isolated from UCB cells treated with detachment reagents Trypsin, Accutase, TrypLE, Collagenase I, Collagenase IV, Dispase II and enzyme-free PBS-based cell dissociation solution had no affect of surface expression of either CD19 or CD133.
(TIFF)

Figure S2 **Kaplin-Meier curves illustrating the proportion of survival of NOD/SCID mice injected with unsorted MCL cells alone (red line) or co-injected with 1×10^6^ irradiated hMSC (green line) or MS-5 cells (black dash line).**
(TIFF)

Figure S3 **Comparison MCL engraftment of NOD/SCID mice injected with unsorted MCL cells alone (red open circle) or co-injected 1×10^6^ irradiated hMSC (green open square) or MS-5 cells (black open triangle).** Engraftment was determined by staining with a hCD45 specific PC7 conjugated and flowcytometric analysis.
(TIFF)

Figure S4 **Engraftment of total (24 mice/sample) NOD/SCID mice injected with of unsorted MCL (Associated with** **Table 3** **).** Each MCL patient is represented by a different symbol.
(TIFF)

Figure S5 **Engraftment of 3° transplant NOD/SCID mice injected with 1×10^6^ CD19+CD133− (red circle) or 500 CD19−CD133+ (black circle) MCL cells (Associated with** **Table 4** **).**
(TIFF)

Figure S6 **CD19−CD133+ MCL cells initiate tumors in NOD/SCID mice (A) A representative example of mice injected with CD19+CD133− (left) and CD19−CD133+ (right) showing abdominal swelling (arrows).** (B) A representative tumor at the time of sacrifice (arrow) 14 weeks post-injection. CD19+CD133− (left) and CD19−CD133+ (right) (C) Comparison on spleen size in CD19+CD133− (left) and CD19−CD133+(right). Arrow points to enlarged spleen from CD19−CD133+ injected mouse.
(TIFF)

Figure S7 **Immunohistochemical analysis of tonsil and CD19−CD133+ xenograft tumors.** Tonsil tissue (control) and tumor tissue were stained with H&E, antibodies specific for human CD3, CD5, CD23, CD19, CD20, CD45, CD79a, CD133, Cyclin D1, Pax5 and FISH analysis for the t(11;14) translocation.
(TIFF)

Figure S8 **Immunohistochemical analysis of tonsil and CD19−CD133+ xenograft tumors.** Tonsil tissue (control) and tumor tissue were stained with H&E, antibodies specific for human CD3, CD5, CD23, CD19, CD20, CD45, CD79a, CD133, Cyclin D1, Pax5 and FISH analysis for the t(11;14) translocation.
(TIFF)

Figure S9 **Immunohistochemical analysis of tonsil and CD19−CD133+ xenograft tumors.** Tonsil tissue (control) and tumor tissue were stained with H&E, antibodies specific for human CD3, CD5, CD23, CD19, CD20, CD45, CD79a, CD133, Cyclin D1, Pax5 and FISH analysis for the t(11;14) translocation.
(TIFF)
