# Supplementary figures and images for: Cobblestone-Area Forming Cells Derived from Patients with Mantle Cell Lymphoma Are Enriched for CD133+ Tumor-Initiating Cells
Source: PLoS One. 2014 Apr 10;9(4):e91042. doi: 10.1371/journal.pone.0091042 (PMC3982953; doi:10.1371/journal.pone.0091042)

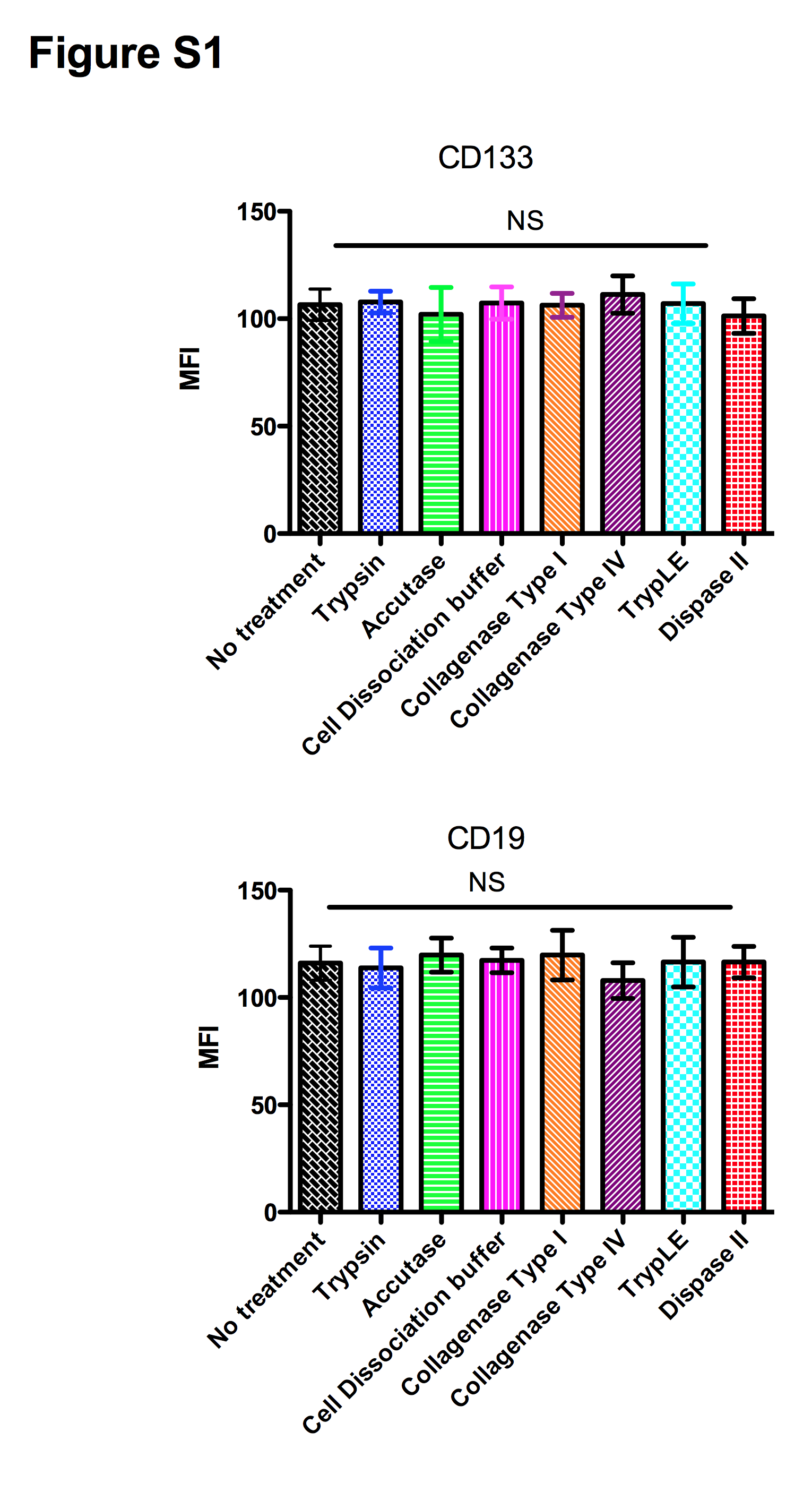

Supplement: Figure S1 — The effects of different Cell detachment reagents on CD19 and CD133 surface expression. CD19+ and CD133+, cells isolated from UCB cells treated with detachment reagents Trypsin, Accutase, TrypLE, Collagenase I, Collagenase IV, Dispase II and enzyme-free PBS-based cell dissociation solution had no affect of surface expression of either CD19 or CD133. (TIFF) [file pone.0091042.s001.tiff]

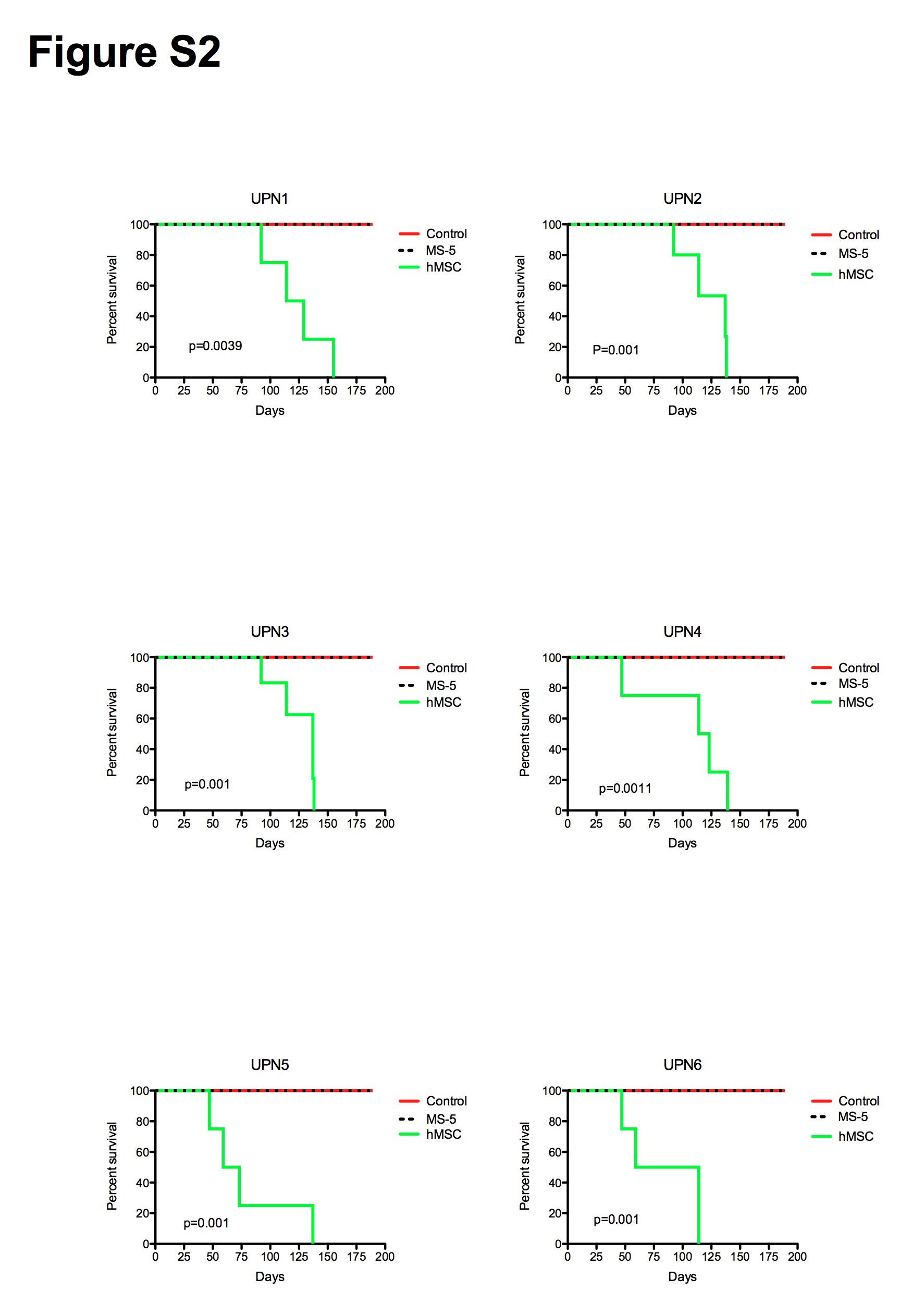

Supplement: Figure S2 — Kaplin-Meier curves illustrating the proportion of survival of NOD/SCID mice injected with unsorted MCL cells alone (red line) or co-injected with 1×10^6^ irradiated hMSC (green line) or MS-5 cells (black dash line). (TIFF) [file pone.0091042.s002.tiff]

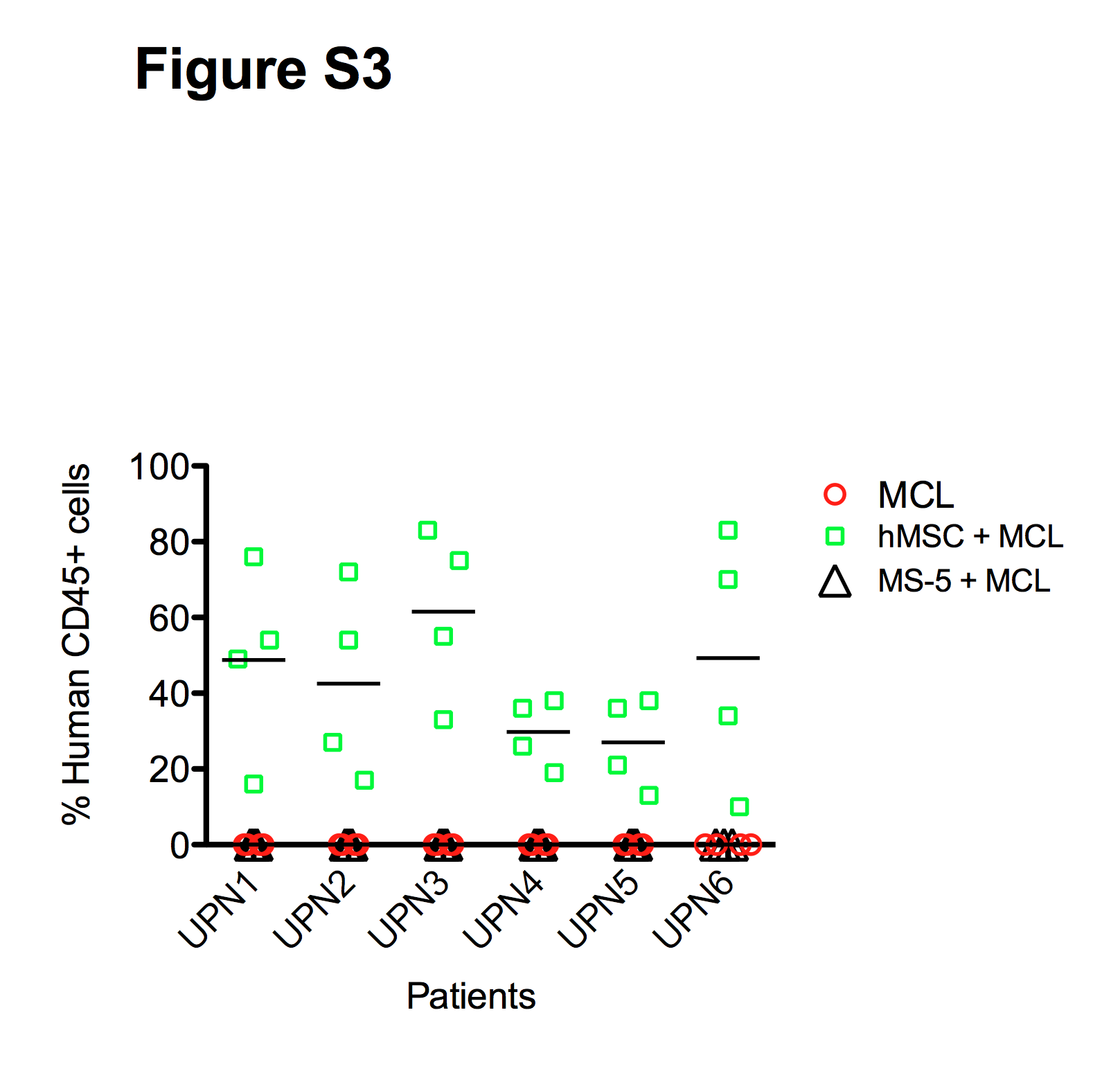

Supplement: Figure S3 — Comparison MCL engraftment of NOD/SCID mice injected with unsorted MCL cells alone (red open circle) or co-injected 1×10^6^ irradiated hMSC (green open square) or MS-5 cells (black open triangle). Engraftment was determined by staining with a hCD45 specific PC7 conjugated and flowcytometric analysis. (TIFF) [file pone.0091042.s003.tiff]

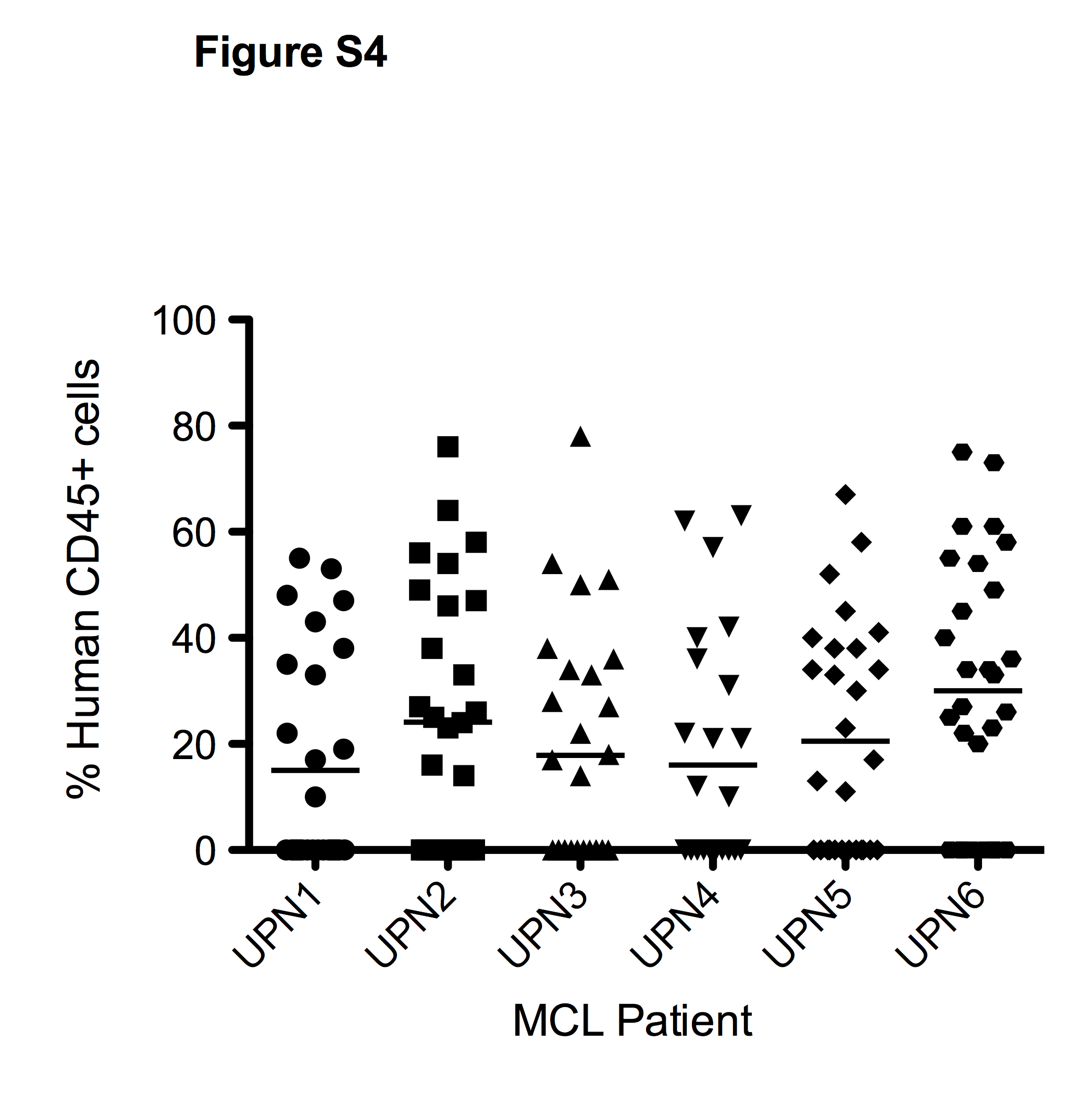

Supplement: Figure S4 — Engraftment of total (24 mice/sample) NOD/SCID mice injected with of unsorted MCL (Associated with Table 3 ). Each MCL patient is represented by a different symbol. (TIFF) [file pone.0091042.s004.tiff]

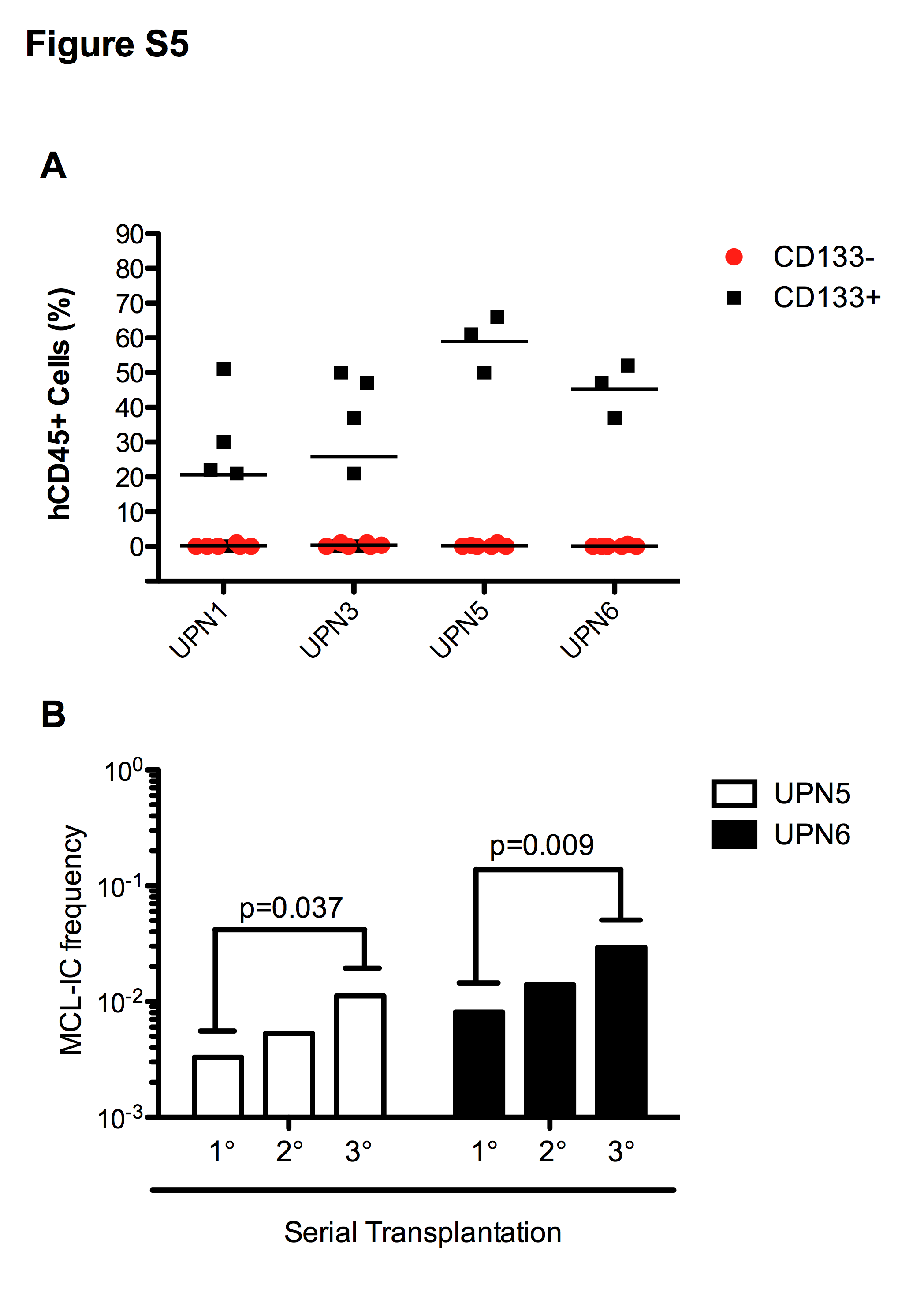

Supplement: Figure S5 — Engraftment of 3° transplant NOD/SCID mice injected with 1×10^6^ CD19+CD133− (red circle) or 500 CD19−CD133+ (black circle) MCL cells (Associated with Table 4 ). (TIFF) [file pone.0091042.s005.tiff]

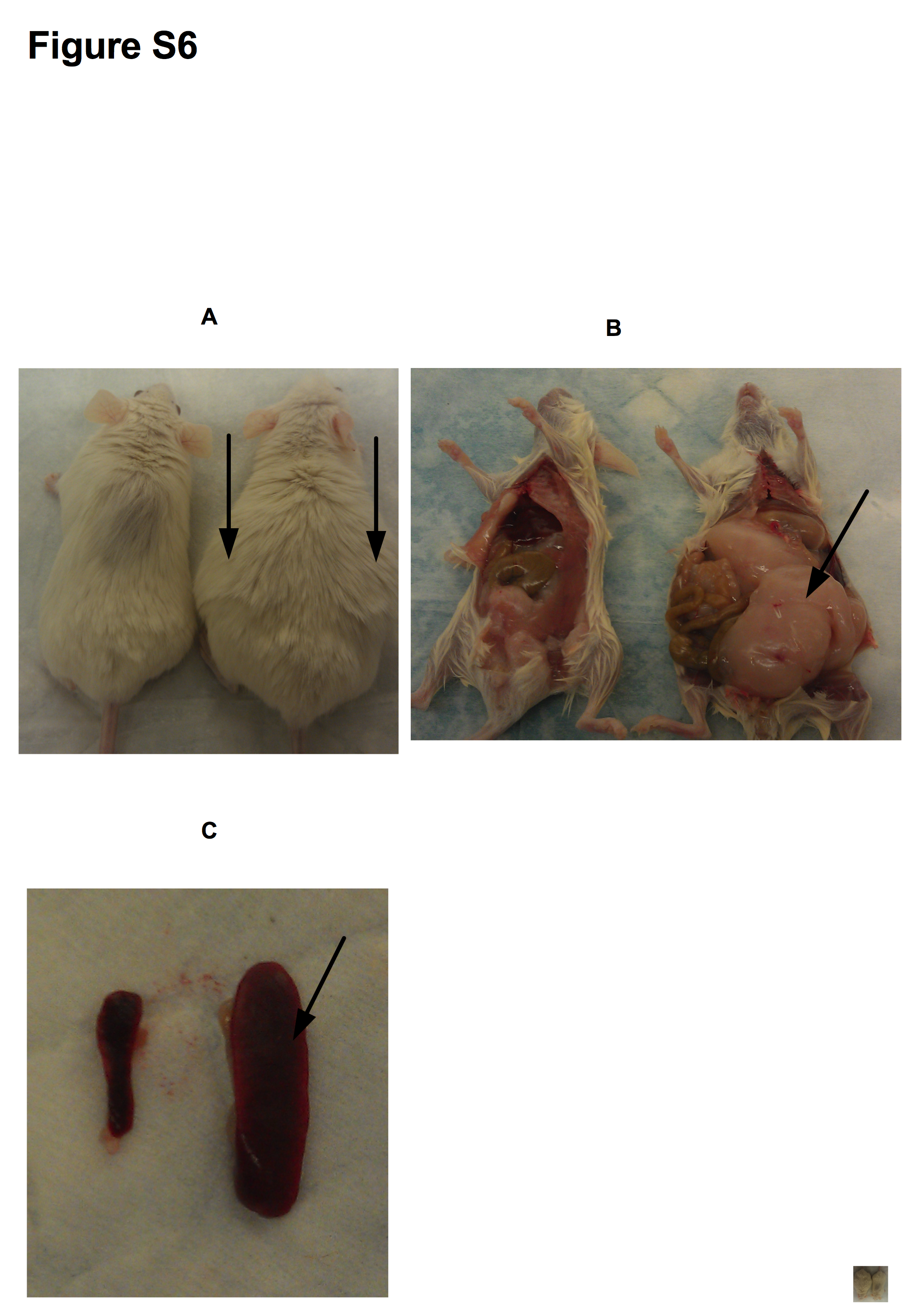

Supplement: Figure S6 — CD19−CD133+ MCL cells initiate tumors in NOD/SCID mice (A) A representative example of mice injected with CD19+CD133− (left) and CD19−CD133+ (right) showing abdominal swelling (arrows). (B) A representative tumor at the time of sacrifice (arrow) 14 weeks post-injection. CD19+CD133− (left) and CD19−CD133+ (right) (C) Comparison on spleen size in CD19+CD133− (left) and CD19−CD133+(right). Arrow points to enlarged spleen from CD19−CD133+ injected mouse. (TIFF) [file pone.0091042.s006.tiff]

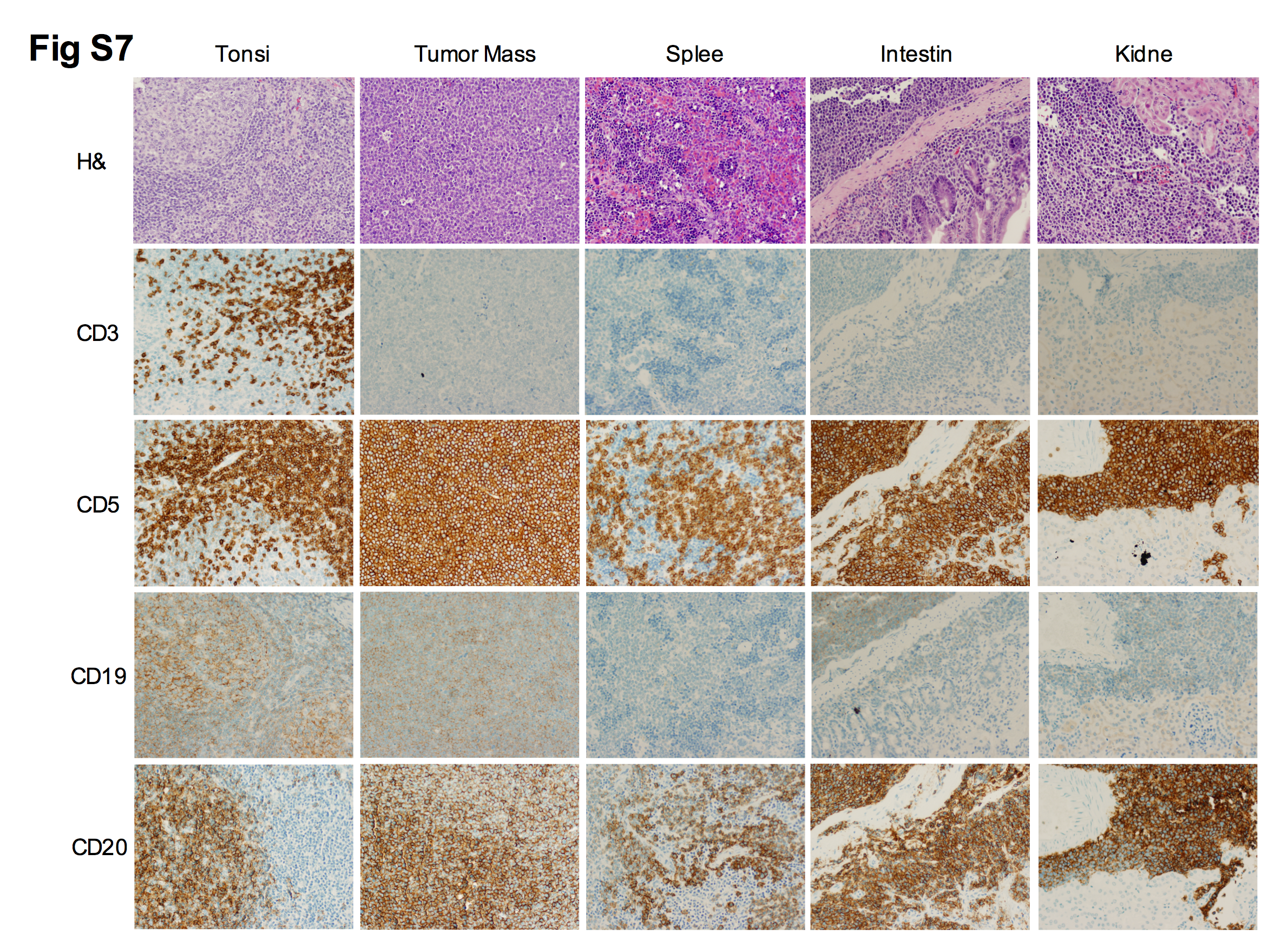

Supplement: Figure S7 — Immunohistochemical analysis of tonsil and CD19−CD133+ xenograft tumors. Tonsil tissue (control) and tumor tissue were stained with H&E, antibodies specific for human CD3, CD5, CD23, CD19, CD20, CD45, CD79a, CD133, Cyclin D1, Pax5 and FISH analysis for the t(11;14) translocation. (TIFF) [file pone.0091042.s007.tiff]

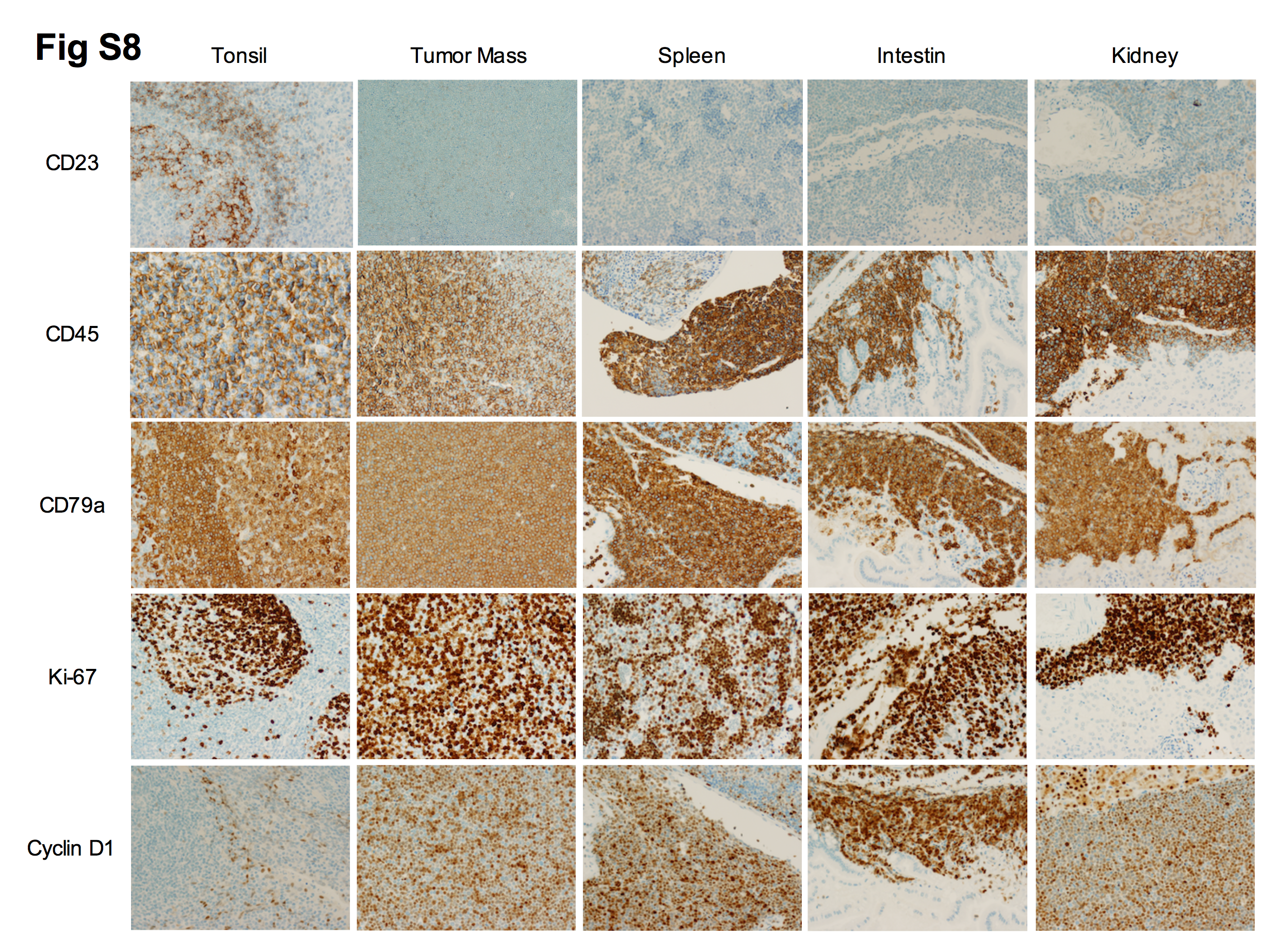

Supplement: Figure S8 — Immunohistochemical analysis of tonsil and CD19−CD133+ xenograft tumors. Tonsil tissue (control) and tumor tissue were stained with H&E, antibodies specific for human CD3, CD5, CD23, CD19, CD20, CD45, CD79a, CD133, Cyclin D1, Pax5 and FISH analysis for the t(11;14) translocation. (TIFF) [file pone.0091042.s008.tiff]

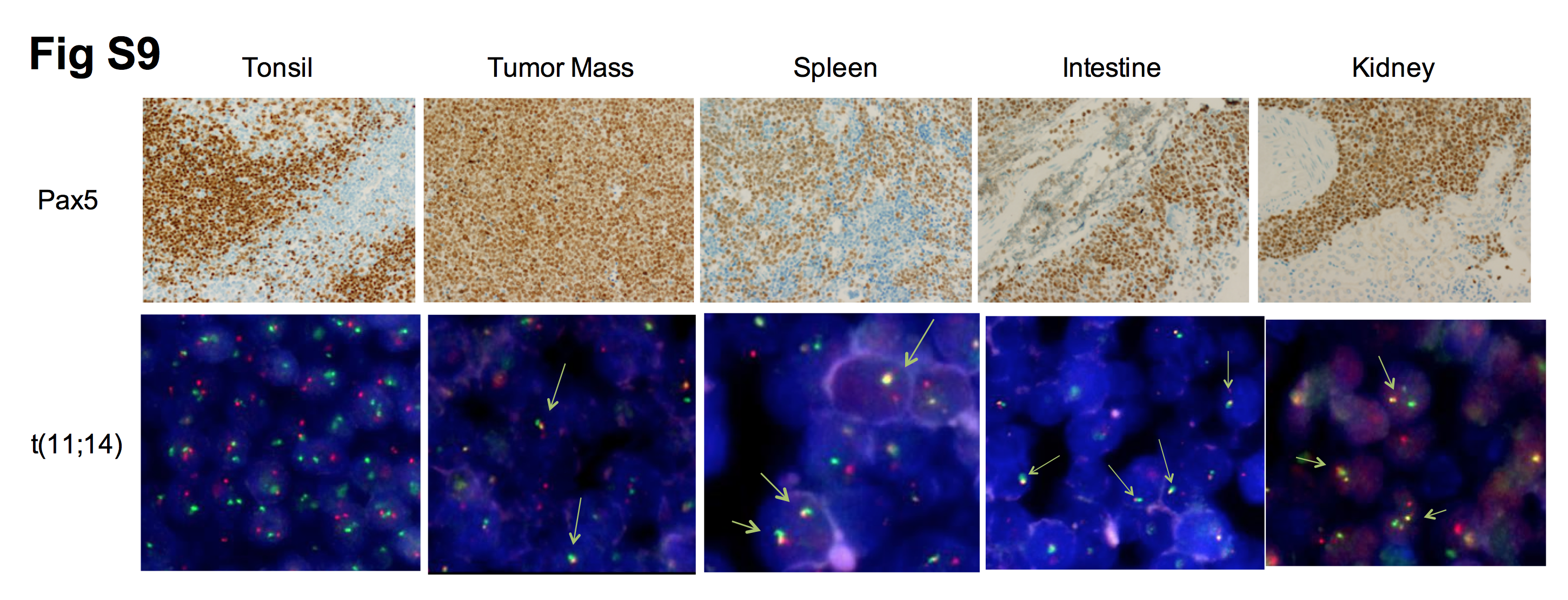

Supplement: Figure S9 — Immunohistochemical analysis of tonsil and CD19−CD133+ xenograft tumors. Tonsil tissue (control) and tumor tissue were stained with H&E, antibodies specific for human CD3, CD5, CD23, CD19, CD20, CD45, CD79a, CD133, Cyclin D1, Pax5 and FISH analysis for the t(11;14) translocation. (TIFF) [file pone.0091042.s009.tiff]
